# Effect of Composition on the Thermo-Induced Aggregation of Poloxamer-Analogue Triblock Terpolymers

**DOI:** 10.1021/acs.macromol.4c02217

**Published:** 2025-02-26

**Authors:** Shaobai Wang, Alberto Alvarez-Fernandez, Xu Liu, Sofia Miron-Barroso, Kelvin Wong, Stefan Guldin, Theoni K. Georgiou

**Affiliations:** † Department of Materials, Royal School of Mines, Imperial College London, London SW7 2AZ, U.K.; ‡ Centro de Fisica de Materiales (CFM) (CSIC-UPV/EHU), Material Physics Centre, Paseo Manuel de Lardizabal 5, San Sebastian 20018, Spain; § Department of Chemical Engineering, University College London, London WC1E 7JE, U.K.; ∥ Department of Life Science Engineering, Technical University of Munich, 85354 Freising, Germany; ¶ TUMCREATE, 1 CREATE Way, #10-02 CREATE Tower, 138602, Singapore

## Abstract

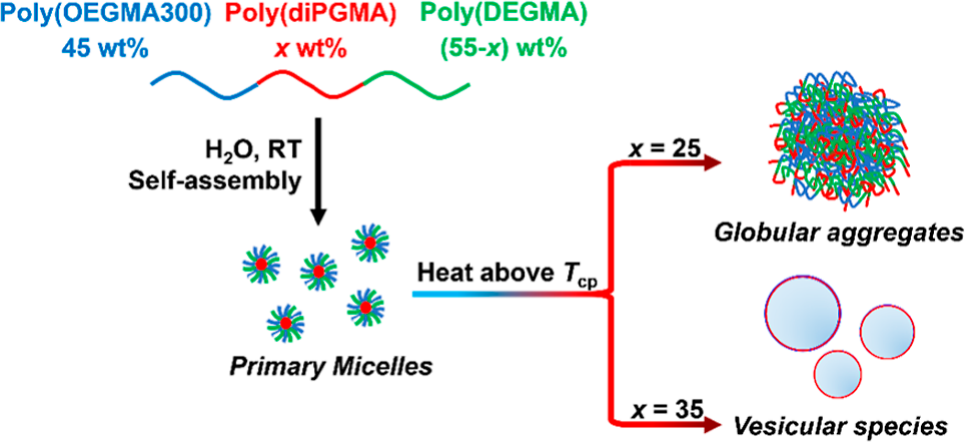

Thermoresponsive polymers hold great promise for biomedical applications due to their thermo-induced phase transitions. However, challenges including controlling transition temperatures, aggregate behavior, or complex synthesis, have limited their broader use. In this study, six ABC triblock terpolymers were synthesized via group transfer polymerization, targeting a molar mass of 8000 g/mol with varying compositions. The terpolymers consist of hydrophilic oligo(ethylene glycol) methyl ether methacrylate (average molar mass = 300 g/mol, OEGMA300), hydrophobic di(propylene glycol) methyl ether methacrylate (diPGMA), and less-hydrophilic di(ethylene glycol) methyl ether methacrylate (DEGMA). Systematic characterizations of properties related to thermo-induced aggregation, including cloud point temperature, aggregate morphology, and chain immobilization, identified a unique dual-stage phase transition in the terpolymer containing 45 wt % OEGMA300, 35 wt % diPGMA, and 20 wt % DEGMA. Instead of directly agglomerating into globular aggregates, this terpolymer transitioned from spherical micelles to vesicular species, offering valuable insights for the design of controllable and responsive polymer systems.

## 1 Introduction

Thermoresponsive polymers with a lower critical solution temperature (LCST) have garnered significant interest in recent decades due to their unique responsiveness to external thermal stimuli.^1−7^ When heated above a specific temperature threshold, these polymers undergo a notable shift in solubility in aqueous media, transitioning from a soluble to an insoluble state, which results in phase transitions such as gelation and precipitation.^7−10^ The LCST-type phase transition is related to a series of hydrophilic and hydrophobic interactions, but from a classic thermodynamic perspective, this process is primarily driven by the entropy change of the water molecules released from the disrupted hydrated shell surrounding the polymer chain at higher temperatures.^7,11−14^ The LCST-type thermoresponsive polymers are considered particularly advantageous in the development of novel biomedical applications, such as controlled drug release, tissue regeneration, and biosensing.^5,8,9,15^

Given that the biomedical utility of the LCST-type thermoresponsive polymers normally relies on their thermo-induced transitions, there is a compelling need to tailor the transition-related properties of the polymers to suit the specific requirements of the biomedical applications. For example, the transition temperature should ideally be aligned with physiological-relevant temperature range.^16−19^ In this objective, one effective strategy consists in the usage of amphiphilic block copolymers. Hence, the balance between the hydrophobic and hydrophilic interactions of the polymers can be adjusted by altering their structural features, such as the chemical composition of the hydrophobic and hydrophilic components, the chain architecture, and the overall molar mass (MM).^20−29^ This strategy has been well demonstrated in the development of poloxamers (Pluronics), a large family of ABA triblock copolymers consisting of hydrophilic poly(ethylene glycol) (PEG, block A) and hydrophobic poly(propyl glycol) (PPG, block B). By varying the structural features of the poloxamers, their thermoresponsive properties, such as the critical micelle temperature (CMT), the cloud point temperature (*T*_cp_), the temperature of gelation (*T*_gel_), the storage modulus (*G*′) of the thermo-induced hydrogel, etc., can be conveniently tailored in order to meet the diverse requirements of various biomedical applications, which has been extensively reported and reviewed.^20−26^

Benefiting from the development of controlled polymerization techniques, a broad variety of monomers has been employed to configure the thermoresponsive amphiphilic block copolymers.^30^ Among them, oligo(ethylene glycol) methyl ether methacrylate (OEGMA) monomers with various side chain lengths have aroused extensive interest.^31−33^ This type of monomers are also considered promising for biomedical applications since they are principally constituted by ethylene glycol (EG) units which are biocompatible and chemically inert.^31,34^ In fact, the modification of drugs with EG units (PEGylation) has achieved significant advances in pharmaceutical development, with more than ten products approved by the U.S. Food and Drug Administration (FDA).^35^ Another significant feature of the OEGMA monomers is the ability to fine-tune the thermoresponsive performance of the corresponding copolymers, which can be achieved by not only the overall structural features of the polymers, but also the length of the EG side chains.^36^ For example, our group reported a series of triblock terpolymers based on *n*-butyl methacrylate (BuMA), 2-(dimethylamino)ethyl methacrylate (DMAEMA), and OEGMA monomers with various side chain lengths.^37^ By increasing the side chain length of the OEGMA monomer from 2, to 5, and to 9 EG units, the *T*_cp_ of the obtained terpolymers increases from 43, to 52, and to 62 °C, respectively. Compared to poly(*N*-isopropylacrylamide) (PNIPAM), the “gold standard”, such flexibility could offer a greater scope for tailoring the thermoresponsive properties.^31^

In a previous study, our group introduced a new strategy to synthesize poloxamer-analogue triblock copolymers by copolymerising the OEGMA monomer with an average side chain length of 4.5 EG units (MM = 300 g/mol, OEGMA300) and an in-house synthesized methacrylate derivative of PPG, di(propylene glycol) methyl ether methacrylate (diPGMA), resulting in ABA-type poly(OEGMA300-*b*-diPGMA-*b*-OEGMA300) triblock copolymers.^29^ The synthesis of these copolymers was achieved through one-pot group transfer polymerization (GTP), a technique that is well-known for its efficacy in producing copolymers with precisely controlled structures, quantitative yield, and rapid kinetics on large scales.^38,39^ Through this approach, the PEG- and PPG-based backbone of the poloxamers was successfully introduced into the side chain of the polymethacrylate-based copolymer. Compared to the original poloxamers which are limited to postfunctionalization on the two end groups, our strategy can be particularly advantageous in the fabrication of poloxamer-derivatives with higher degree of functionalization since the monomers with functional structures, such as fluorescent groups and “clickable” groups, can be easily introduced into the polymer chains in a proper stage of the GTP process.

Continuing from the previous studies on the poloxamer-analogue triblock copolymers, a new series of six ABC triblock terpolymers consisting of the hydrophilic OEGMA300 (unit A), the hydrophobic diPGMA (unit B), and the less-hydrophilic di(ethylene glycol) methyl ether methacrylate (DEGMA, unit C) were prepared via the same GTP technique. The selection of DEGMA as the third comonomer was inspired by one of our another reports on poly(OEGMA300-*b*-BuMA-*b*-DEGMA), which demonstrated gelling ability from an impressively low concentration of 2 wt %.^27^ The overall MM of the six terpolymers was controlled consistently at approximately 8000 g/mol, while the compositions were systemically varied. To the best of our knowledge, the chemistry of the polymers presented here has not been previously reported. The key focus of this study lies in the effect of the polymer composition on the properties related to the thermo-induced aggregation, such as the *T*_cp_, the dimension and structure of the aggregates, and chain immobilization. To achieve this, a comprehensive investigation was taken, using multiple techniques including ultraviolet–visible spectroscopy (UV–vis), dynamic light scattering (DLS), transmission electron microscopy (TEM), variable-temperature proton nuclear magnetic resonance spectroscopy (VT-^1^H NMR), and small-angle X-ray scattering (SAXS), under various temperatures below and above the respective *T*_cp_.

## 2 Experimental Section

### 2.1 Materials

The following chemical compounds were purchased from Sigma-Aldrich UK, including: OEGMA300 (95%, contains 100 ppm MEHQ as inhibitor), di(propylene glycol) monomethyl ether (diPGOH, mixture of isomers, > 95%), methacryloyl chloride (97%, contains 200 ppm MEHQ), DEGMA (95%, contains 100 ppm MEHQ), methyl trimethylsilyl dimethylketene acetal (MTS, 95%), Et_3_N (anhydrous, 99%), THF (anhydrous, inhibitor-free), 2,2-diphenyl-1-picrylhydrazyl (DPPH), calcium hydrate (90%, powder), dimethyldichloridesilane (99.5%), basic alumina, pyrene (98%), and poly(methyl methacrylate) (PMMA) standard samples for GPC calibration (2, 4, 8, 20, 50, and 100 kg/mol). Acetone (>99%), ethanol absolute, and *n*-hexane (>98%) were from Fisher Scientific (UK). Uranyl acetate for negative staining was from Honeywell (UK). The catalyst for the polymerization, tetrabutyl ammonium bibenzoic acid (TBABB), was prepared before this study according to Dicker et al.^40^

### 2.2 Monomer Synthesis and Purification

The synthesis of diPGMA followed the same protocol in our previous research and is described as follows:^29^ diPGOH, THF, Et_3_N, and methacryloyl chloride (molar ratio of 1:2:4:1.2), along with ∼10 mg of DPPH, were added into a two-liter flask and stirred magnetically overnight in an ice bath. After filtration to remove the Et_3_N·HCl byproduct, the filtrate was passed through a basic alumina column twice to remove any acidic residuals. The diPGMA monomer was then obtained by distilling the filtrate and stored with CaH_2_ and DPPH. ^1^H NMR of diPGMA: (400 MHz, chloroform-*d*): δ 5.86 (s, 1H), 5.31 (s, 1H), 4.94–4.80 (m, 1H), 3.43–3.05 (m, 8H), 1.71 (s, 3H), 1.71–1.03 (dd, *J* = 6.4, 2.4 Hz, 3H), 0.90–0.88 (m, 3H). The ^1^H NMR spectrum of the obtained diPGMA is shown in Figure S1 and agrees well with the previous research.^29^ For other compounds, DEGMA was purified by passing it through the basic alumina column twice to remove MEHQ inhibitor before being stored with CaH_2_ and DPPH, while OEGMA300 was prepared as 50–50 vol % solution in THF, treated with the basic alumina column and stored with CaH_2_ only. The catalyst, TBABB, was dried under vacuum overnight before use.

### 2.3 Group Transfer Polymerization of the Terpolymers

The terpolymers were prepared via the one-pot GTP technique. Before the reaction, OEGMA300 was directly filtered through a syringe filter, while all the other liquid compounds, including diPGMA, DEGMA, and MTS, were distilled to remove DPPH and CaH_2_. In a typical GTP process, ∼ 10 mg of TBABB was added into a 250 mL hydrophobized round-bottom flask, followed by purging with argon and the addition of THF and MTS. The polymerization was then started by sequentially adding the monomers, i.e., OEGMA300, diPGMA, and DEGMA, into the system. Each monomer (block) was allowed to react for 15 min and ∼0.2 mL sample was collected before the next monomer was added. After the polymerization of the final block, the reaction was quenched by 1 mL of ethanol. The terpolymers were purified by direct precipitation in *n*-hexane and dried under vacuum for 30 days until a constant weight was reached.

### 2.4 Characterizations

#### 2.4.1 Gel Permeation Chromatography

The MM of the obtained terpolymers and their precursors are determined by an Agilent 1260 infinity GPC system (Agilent, US) equipped with a refractive index (RI) detector The mobile phase is THF-Et_3_N (95–5% v/v) mixture running at 1.0 mL/min and the temperature of the system was 30 °C. Before the measurements, the instrument was calibrated by standard PMMA samples at 2, 4, 8, 20, 50, and 100 kg/mol.

#### 2.4.2 Nuclear Magnetic Resonance Spectroscopy

All the nuclear magnetic resonance (NMR) spectra were recorded on a JEOL ECZ 400S spectrometer (400 MHz, JEOL, Japan) To characterize the chemical structures of the obtained polymers, the ^1^H NMR tests were conducted at 25 °C with 16 scans, using samples dissolved in CDCl_3_. For the variable-temperature proton NMR (VT-^1^H NMR), the tests were performed with 1 wt % polymer solutions in D_2_O. Before each VT test, samples were equilibrated at the testing temperature for 5 min, with a relaxation time of 10 and 16 scans.

#### 2.4.3 Dynamic Light Scattering

Dynamic light scattering (DLS) tests were performed in triplicate on a Zetasizer Nano ZSP (Malvern, UK) with a laser wavelength of 632.8 nm at a backscattering angle of 173°. Before each measurement, the sample was filtered through a syringe filter and equilibrated for 300 s at the testing temperature. The acquired data was processed on Zetasizer software 8.02.

#### 2.4.4 Pyrene Fluorometric Analysis

To prepare the sample solutions, ∼10 μL of dilute pyrene stock solution in acetone was added into a 3.5 mL glass vial, followed by blow-drying under filtered argon flow. The obtained pyrene-containing vials were then used to prepare the test solutions by diluting a polymer aqueous stock solution into desired concentrations (varying from 0.1 mM to 0.02 μM) within them. The fluorescence emission spectra of pyrene were recorded with an excitation at 334 nm (excitation and emission slits were both 3.0 nm) on a PerkinElmer LS55 fluorometer (PerkinElmer, US) at 25 °C during three independent measurements.

#### 2.4.5 Ultraviolet–Visible Spectroscopy (UV–vis)

Transmittance-temperature relationships of the polymer solutions were recorded on an Agilent Cary Compact Peltier UV–vis spectrometer (Agilent, US) under a testing wavelength of 550 nm. The sample was contained in a 10 mm quartz cuvette, treated with magnetic stirring and slowly heated up at 0.2 °C/min. When reporting the results, the absorbance (*A*) was converted to transmittance (*T*) by
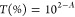
1

#### 2.4.6 Transmission Electron Microscopy

The morphological micrographs of the polymeric aggregates were recorded using a JEOL STEM 2100Plus transmission electron microscope (JEOL, Japan), operating on an accelerating voltage of 200 kV Samples were prepared in an oven (Genlab, UK) with controlled temperature and the general protocol is described as follows: a drop (∼8 μL) of the polymer solution was deposited on an AGS160H grid (Agar scientific, UK) which was irradiated in UV light for 1 h in advance. The drop of solution was blotted by a piece of filter paper after depositing for 1 min, followed by washing and a second blotting. The sample-loaded grid was then negatively stained by a drop of 2 wt % uranyl acetate solution, blotted, and dried. Size analysis was performed with ImageJ 1.54f.

#### 2.4.7 Small Angle X-ray Scattering

Small angle X-ray scattering (SAXS) experiments were performed on a Ganesha 300XL (SAXSLAB, US) coupled with a high brilliance microfocus Cu source (λ = 1.54 Å) and a Linkam capillary HFXS350-CAP stage. Before the measurements, the beam center and the sample-to-detector distance were calibrated using the diffraction pattern of silver behenate standard. The samples were sealed into borosilicate glass capillaries with hot glue before being loaded into the measurement chamber. For each test, the samples were equilibrated for 300 s at the testing temperature and the exposure time was 1800 s. 2D SAXS patterns were collected by a Pilatus 300 K solid-state photon-counting detector with a 2 mm beam stop and a sample-to-detector distance of 950 mm. The obtained 2D patterns were then radially averaged around the direct beam position using the SAXSGUI software to 1D SAXS profiles, which were modeled in SasView 5.0.6 software.^41^ Detailed information on the models and results derived can be found in Supporting Information.

## 3 Results and Discussion

In this section, the chemical structure of the obtained terpolymers was characterized by GPC and ^1^H NMR, while the thermo-induced phase transition behaviors of the terpolymers in deionized (DI) water were investigated via multiple techniques, including UV–vis, DLS, TEM, VT-^1^H NMR, and SAXS.

### 3.1 Synthesis of the Triblock Terpolymers

**Figure 1:**
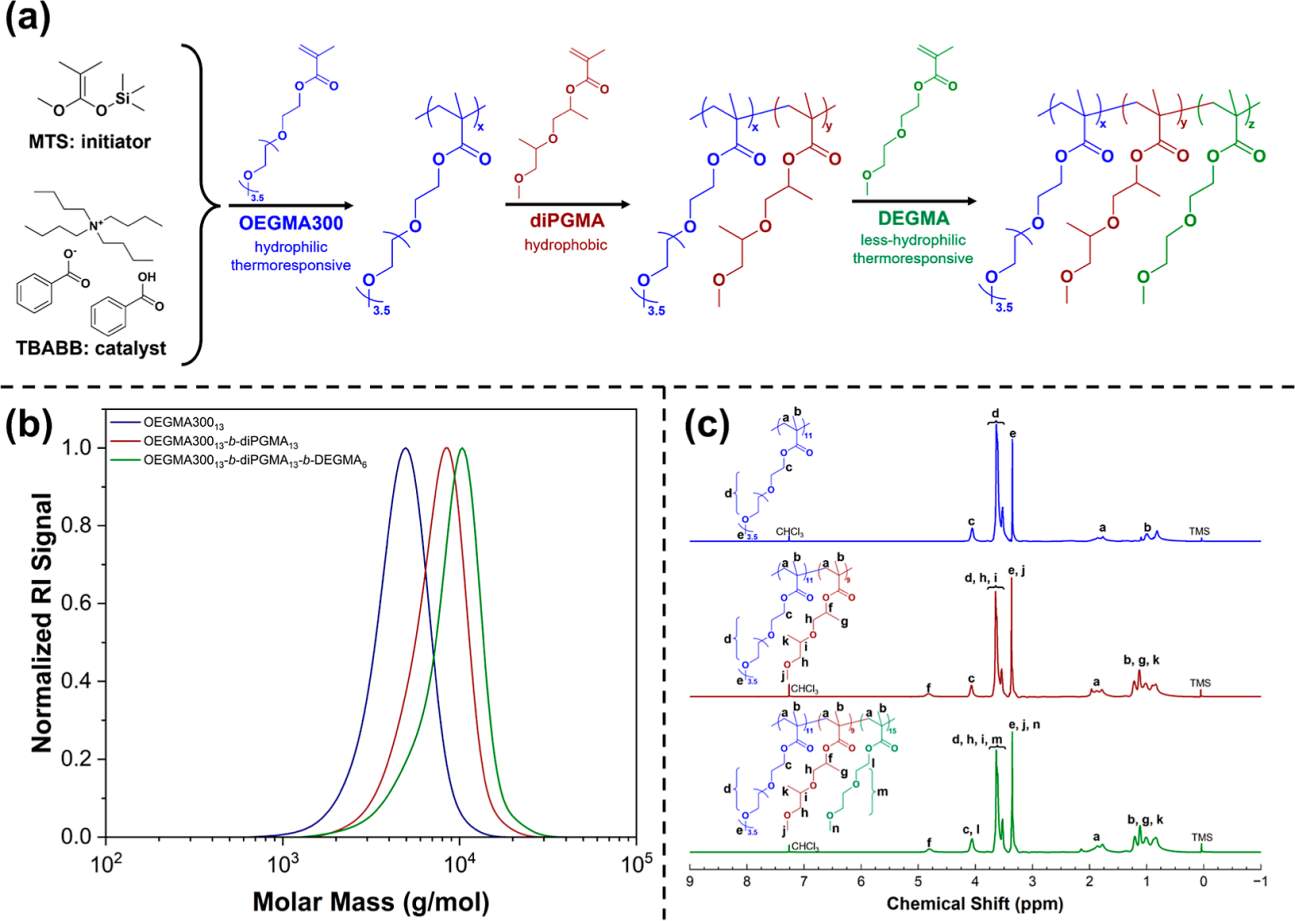
(a) Reaction route of the GTP technique involved in this study, (b) GPC traces of the terpolymer OPD(50-35-15) and the precursors in THF/Et_3_N 95/5 vol % mixture, and (c) ^1^H NMR spectra of the terpolymer OPD(50-35-15) and the precursors in CDCl_3_ at 25 °C. For conciseness, only the most abundant isomer of diPGMA is shown.^29^

As illustrated in Figure 1a, the triblock terpolymers were synthesized via the one-pot GTP technique, in which MTS and TBABB served as the initiator and the catalyst, respectively. The monomers, OEGMA300, diPGMA, and DEGMA, were sequentially injected into the reaction system to configure the triblock terpolymers. In this study, two series of terpolymers were synthesized, each with a consistent MM at approximately 8000 g/mol but different OEGMA300 contents at 45 and 50 wt %. In each series, three terpolymers are included, varying in diPGMA contents at 25, 30, and 35 wt %. For clarity, the terpolymers are named after their chemical compositions. For example, the terpolymer with a composition of 45 wt % OEGMA300, 25 wt % diPGMA, and 30 wt % DEGMA is named as OPD(45-25-30), where “O”, “P”, and “D” are the abbreviations of OEGMA300, diPGMA, and DEGMA, respectively.

To confirm the chemical structure of the obtained terpolymers, GPC and ^1^H NMR were employed to determine the MM and composition of the obtained terpolymers in a block-by-block approach. The GPC traces and ^1^H NMR spectra of terpolymer OPD(50-35-15) are presented in Figure 1b,c, respectively, as an example. In Figure 1b, the GPC trace moves progressively from lower to higher MM region as the polymer chain propagates from the homopolymer [poly(OEGMA300)_13_], through the diblock copolymer [poly(OEGMA300_13_-*b*-diPGMA_13_)], and to the final triblock terpolymer [poly(OEGMA300_13_-*b*-diPGMA_13_*b*-DEGMA_6_)]. For the final product, the MM determined from GPC is close to the theoretical target, exhibiting a narrow distribution with a low dispersity index (D̵) of about 1.2, as detailed in Table 1. Moreover, the absence of any tails or secondary shoulders in the GPC traces indicates a high conversion rate of the precursory species. This feature is consistent with the other five terpolymers, as shown in Figure S2.

**Table 1:** Theoretical Structures, Target Molar Mass (MM^Target^), Number-Average Molar Mass (*M*_n_) and Dispersity Index (D̵) Measured by GPC, and Theoretical Composition and Experimental Results Derived from the ^1^H NMR Spectra of the Obtained Polymers

sample no	theoretical structure	MM^Target^^a^ (g/mol)	*M*_n_ (g/mol)	D̵	OEGMA-diPGMA-DEGMA (wt %)	
					theoretical	^1^H NMR
OPD (45-25-30)	O_12_	3655	4100	1.14	100-0-0	100-0-0
	O_12_-*b*-P_9_	5630	5600	1.12	64-36-0	65-35-0
	O_12_-*b*-P_9_-*b*-D_13_	8000	7900	1.12	45-25-30	46-25-29
OPD (45-30-25)	O_12_	3655	4100	1.15	100-0-0	100-0-0
	O_12_-*b*-P_11_	6025	6300	1.14	60-40-0	61-39-0
	O_12_-*b*-P_11_-*b*-D_11_	8000	8200	1.15	45-30-25	46-29-25
OPD (45-35-20)	O_12_	3655	3900	1.17	100-0-0	100-0-0
	O_12_-*b*-P_13_	6420	6400	1.19	56-44-0	58-42-0
	O_12_-*b*-P_13_-*b*-D_8.5_	8000	8100	1.21	45-35-20	46-34-20
OPD (50-25-25)	O_13_	4050	4600	1.14	100-0-0	100-0-0
	O_13_-*b*-P_9_	6025	6200	1.13	67-33-0	67-33-0
	O_13_-*b*-P_9_-*b*-D_11_	8000	8100	1.13	50-25-25	50-25-25
OPD (50-30-20)	O_13_	4050	4900	1.13	100-0-0	100-0-0
	O_13_-*b*-P_11_	6420	7400	1.14	63-37-0	63-37-0
	O_13_-*b*-P_11_-*b*-D_8.5_	8000	8700	1.17	50-30-20	51-29-20
OPD (50-35-15)	O_13_	4050	4200	1.16	100-0-0	100-0-0
	O_13_-*b*-P_13_	6815	6900	1.16	59-41-0	59-41-0
	O_13_-*b*-P_13_-*b*-D_6_	8000	8200	1.18	50-35-15	51-35-14

a MM^Target^ was calculated by MM^Target^ = ∑_i_(DP_i_ × MM_i_) + 100, where MM_i_ is molar mass of each monomer, DP_i_ is the degree of polymerization of each block, and 100 is the molar mass of the residual group from MTS (the initiator) after dissociative GTP.

In the ^1^H NMR spectra of OPD(50-35-15), all the peaks can be assigned according to the theoretical chemical structure of the terpolymer, while the absence of C=CH_2_ proton peaks around 5.5 and 6.0 ppm indicates a high conversion rate of the monomers. The chemical composition of the terpolymer was determined from the integral intensity of the COOCH peak of the poly(diPGMA) block (peak f, 4.8 ppm) and the COOCH_2_ peak of the poly(OEGMA300) and poly(DEGMA) blocks (the overlapping peak c and l, 4.1 ppm), which aligns well with the theoretical targets. The MM and chemical composition of all the obtained terpolymers, including their precursors, are summarized in Table 1. As confirmed by GPC and ^1^H NMR, six terpolymers with well-controlled MM and composition were obtained and met the requirements for the scope of this study.

### 3.2 Self-Assembly and Micellization in DI Water

Driven by the tendency to minimize the interfacial energy, amphiphilic block copolymers can naturally self-assemble in aqueous media, forming well-organized structures such as micelles.^42^ To confirm whether this process can take place in the condition of interest, i.e., 1 wt % in DI water from 25 °C, the critical micelle concentration (CMC) of the terpolymers was determined at 25 °C via pyrene fluorometry. When the polymer concentration increases, the interaction between pyrene molecules and the nonpolar hydrophobic segments of the polymer becomes stronger, leading to a gradual decrement in the intensity ratio of the first to the third characteristic peak, *I*_1_/*I*_3_, in the emission spectrum of pyrene.^43,44^ Therefore, the CMC can be determined from the inflection point of the concentration-*I*_1_/*I*_3_ plot (Figure S3).^43^ The obtained CMC values at 25 °C, as listed in Table 2, are all below 2 μM (∼0.002 wt %), evidencing that all the terpolymers are able to self-assemble into micelles at 1 wt % in water. It is also noticed that the CMC is influenced by the composition of the terpolymers. More specifically, the CMC becomes lower by increasing the diPGMA content, which is within the expectation for the formation of a more stable micelle core.^20,45−48^

**Table 2:** Summary of Aqueous Properties of the Investigated Terpolymers, Including the CMC at 25 °C in H_2_O, the Theoretical and Experimental *d*_h_ in 1 wt % Polymer Solution in H_2_O, and *T*_cp_ of the Polymers at 1 wt % in H_2_O

sample no	CMC at 25 °C (μM)^a^	hydrodynamic diameter (*d*_h_) at 25 °C^b^				*T*_cp_ (±0.1 °C)^c^
		by theory (nm)	by intensity (±1 nm)	by number (±1 nm)	PDI	
OPD(45-25-30)	0.33 ± 0.05	8.6	13	9	0.10	43.8
OPD(45-30-25)	0.25 ± 0.07	9.2	14	10	0.06	42.8
OPD(45-35-20)	0.21 ± 0.05	9.5	16	12	0.06	41.7
OPD(50-25-25)	0.75 ± 0.17	9.4	13	9	0.10	47.9
OPD(50-30-20)	0.27 ± 0.07	10.3	15	11	0.06	46.4
OPD(50-35-15)	0.21 ± 0.02	10.5	16	12	0.07	45.3

a The values of CMC were determined at 25 °C by pyrene fluorometry, with an excitation wavelength of 334 nm.

b The values of theoretical *d*_h_ were obtained from either *d*_h_ = 0.254 × (DP_diPGMA_ + 2 × DP_OEGMA300_) or *d*_h_ = 0.254 × (DP_diPGMA_ + 2 × DP_DEGMA_), depending on which hydrophilic block possesses a higher DP. 0.254 (nm) is the projection length of the backbone in a methacrylate repeating unit. The experimental values reported are the mean of the peak values for the size distribution from three repeats. The polydispersity index (PDI) is for the distribution of *d*_h_ by intensity, averaged from three repeats.

c The values of *T*_cp_ were determined by UV–vis spectroscopy under a testing wavelength of 550 nm and a heating rate of 0.2 °C/min. Each test was performed in triplicate.

The micellization of the obtained terpolymers at 1 wt % in DI water was investigated by DLS at 25 °C. For this purpose, the experimental results of hydrodynamic diameter, *d*_h_, were compared to the theoretical values derived from a spherical core–shell model, in which the poly(OEGMA300) block and poly(DEGMA) block constitute the hydrated shell, whereas the poly(diPGMA) block forms the core (Figure S4). To estimate the theoretical *d*_h_ from the core–shell model, three assumptions were involved: (a) the polymer chains are fully extended, (b) the hydrophobic chains in the core are overlapped, and (c) the long side chains of the poly(OEGMA300) block display a negligible impact on the overall *d*_h_. From the experimental results listed in Table 2, both the intensity-averaged and number-averaged *d*_h_ fit well to the theoretical estimates, indicating that the terpolymers are likely to self-assemble into the core–shell micelles at 1 wt % in H_2_O. However, it is worth mentioning that the intensity-averaged *d*_h_s are still slightly higher than the theoretical ones in all cases, even under the assumption that the polymer chains are fully extended. As observed previously, this feature can be attributed to the contribution from the lengthy side chain of poly(OEGMA300) group (approximately 4 nm in total).^27,29^ The size homogeneity of the micelles is also supported well by the low PDI ranging from 0 to 0.1.^28^ Detailed histograms of *d*_h_ by intensity and by number are shown in Figures S5 and S6, respectively.

**Figure 2:**
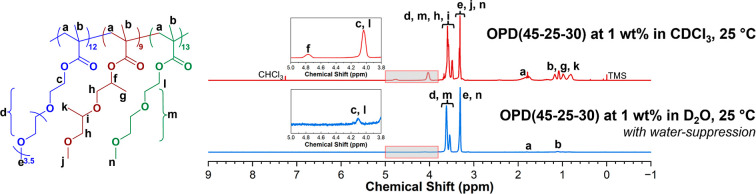
A comparison of ^1^H NMR spectra of OPD(45-25-30) at 1 wt % in CDCl_3_ (top) and D_2_O (bottom, water-suppressed).

The self-assembly structure of the terpolymers was also determined by ^1^H NMR in D_2_O. In such a spectrum, the integral intensity (area) of a given peak is strongly related to the mobility of the corresponding proton.^49−52^ Therefore, the immobilization of the structure can be analyzed by the attenuation of its peak. Figure 2 shows the comparison of ^1^H NMR spectra of OPD(45-25-30) at 1 wt % in CDCl_3_ and D_2_O at 25 °C as an example.

In the spectrum of the D_2_O solution, the characteristic peak f (COOCH group) of the hydrophobic poly(diPGMA) block at ∼4.8 ppm disappears, evidencing that the poly(diPGMA) block is highly restricted and immobilized in the micelle core. This observation further implies that the contribution of poly(diPGMA) signals to other overlapping peaks in the spectrum should be negligible. The signals from the protons on the polymer backbone (peak a and b, 0.8–2.2 ppm) and the COOCH_2_ groups of the poly(OEGMA300) and poly(DEGMA) blocks (peak c and l, 4.2 ppm) are also attenuated, suggesting partial immobilization due to their hydrophobic or less-hydrophilic nature. Conversely, the peaks of the OCH_2_CH_2_ (EG groups) protons and terminal OCH_3_ protons from the side chains of the hydrophilic poly(OEGMA300) and poly(DEGMA) blocks remain well observable, aligning with the expectation that these moieties should act as the hydrated and mobile shell of the micelle. Overall, the ^1^H NMR results strongly support the formation of core–shell micelles.

### 3.3 Thermo-Induced Phase Transition and Aggregation

#### 3.3.1 Turbidity Measurements

The phase transition of the terpolymers at 1 wt % in H_2_O was inspected via multiple techniques, including UV–vis, DLS, TEM, ^1^H NMR, and SAXS. The characterizations were performed at various temperatures ranging from 25 to 55 °C to examine the aggregation behaviors in the pre- and post-transition stage.

**Figure 3:**
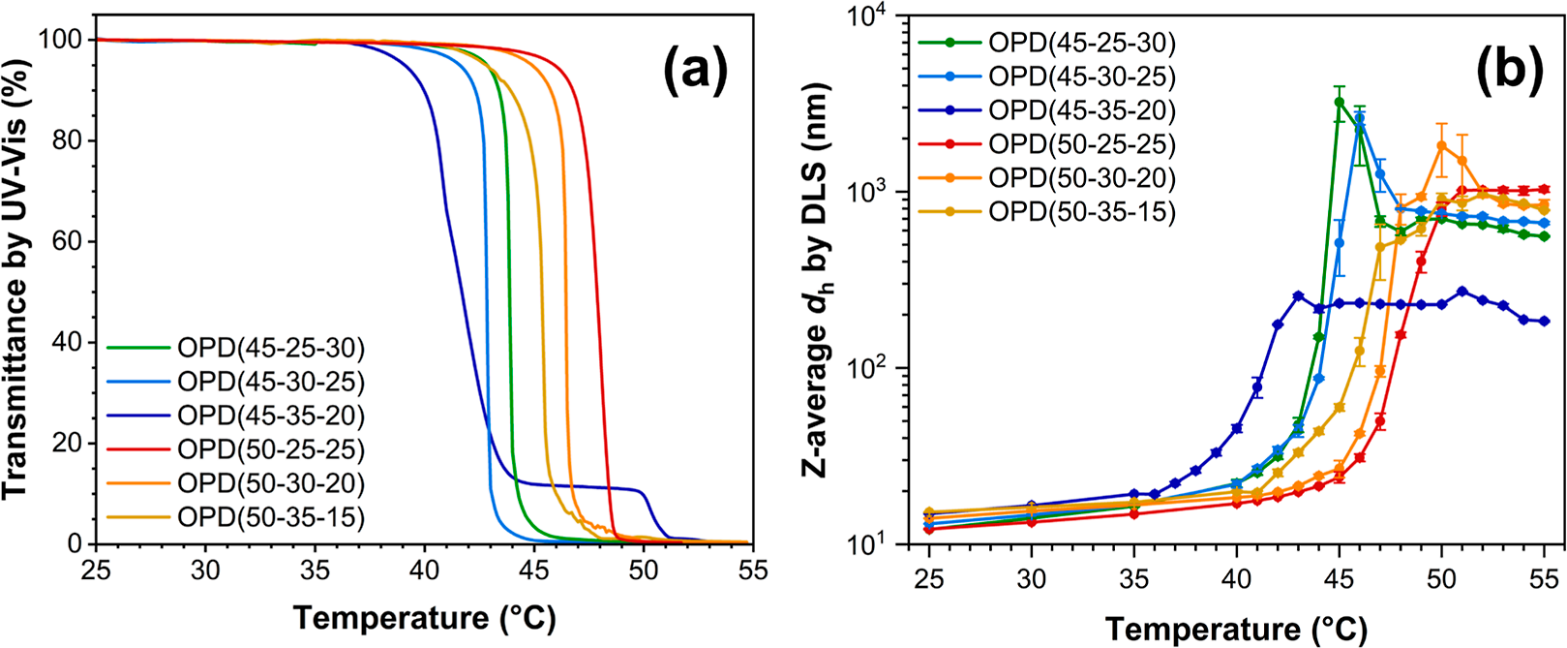
(a) Variation of transmittance, as a function of temperature, of the terpolymers at 1 wt % in H_2_O under a testing wavelength of 550 nm and a heating rate of 0.2 °C/min, and (b) the evolution of Z-average *d*_h_ of the terpolymers at 1 wt % in H_2_O from 25 to 55 °C.

Two types of phase transitions are noticed in the UV–vis spectra (Figure 3a). First, the terpolymer OPD(45-35-20) exhibits a dual-stage transition characterized by an initial decline in transmittance from 100% to about 10% within the range of 38 to 45 °C, followed by a plateau stage from 45 to 50 °C, and eventually a decrement to 0% upon further heating. In contrast, the other five terpolymers, namely OPD(45-25-30), OPD(45-30-25), OPD(50-25-25), OPD(50-30-20), and OPD(50-35-15), show a rapid decline in transmittance from 100% to 0% once heated above a specific temperature threshold. The cloud point temperature (*T*_cp_) was determined from the respective UV–vis spectrum as the temperature point at which the transmittance of the solution decreased to 50%,^53^ as listed in Table 2. Notably, when the OEGMA300 content is held constant, the *T*_cp_ decreases with the increase of the diPGMA content, consistent with previous reports that an increase in the hydrophobic content could lower the *T*_cp_.^29,37,54,55^ Conversely, when the diPGMA content remains constant, the *T*_cp_ increases as the OEGMA300 content increases. This is also expected since increasing the OEGMA300 content would enhance the hydrophilicity of the polymer chain and the stability of the micelles, thereby preventing the thermo-induced aggregation and leading to a higher *T*_cp_.

#### 3.3.2 Micellar Aggregation

The thermo-induced aggregation of the polymeric micelles was investigated using DLS, with the overall intensity-based *d*_h_ distributions at various temperatures shown in Figure S7. For clarity in the comparison, the *Z*-average *d*_h_ was selected to represent the overall intensity-based *d*_h_ distribution, as demonstrated in Figure 3b. Generally, the five terpolymers showing a rapid phase transition exhibit similar aggregation behaviors to each other, despite having different *T*_cp_s. Below the respective *T*_cp_, a marginal increase in the *Z*-average *d*_h_ of the polymer micelles is observed from 25 °C. As the solution is heated near the *T*_cp_, this *d*_h_ increment becomes more pronounced. Taking OPD(45-25-30) as an example, the *Z*-average *d*_h_ increases slightly from approximately 12 nm at 25 °C to about 20 nm at 40 °C, and significantly to about 50 nm at 43 °C (1 °C below its *T*_cp_) with a broad distribution extending to approximately 200 nm (Figure S7). These features suggests that a low extent of micellar aggregation occurs prior to the macroscopic phase transition, even though the primary micelles may still be the predominant species in the solution.

Upon heating slightly above the respective *T*_cp_, the *Z*-average *d*_h_ escalates to micrometre level due to the emergence of larger aggregates (Figures 3b and S7). Consequently, the *d*_h_ distribution becomes multimodal, with the PDI escalating in this temperature zone (Figure S8). Upon further heating to 2–3 °C above the respective *T*_cp_, the unimodal distribution of *d*_h_ is restored. The other four terpolymers exhibiting the rapid phase transition present similar aggregation behaviors both below and above the respective *T*_cp_, as can be found in Figures 3b, S7 and S8. This feature suggests that the core–shell micelles formed by these terpolymers may subsequently follow the same pattern of thermo-induced aggregation. In some cases, a moderate decrease in *d*_h_ is observed (Figure 3b), suggesting that the aggregates are loosely packed at first, followed by water expulsion during the characterization period.^56^

As for the terpolymer OPD(45-35-20) which undergoes a dual-stage phase transition, the micellar aggregation below the *T*_cp_ is similar to the counterparts of the five terpolymers showing a rapid transition (Figures 3b and S7). When heated above the *T*_cp_, the *Z*-average *d*_h_ reaches approximately 230 nm in 45–50 °C, which is significantly lower than those of the other five terpolymers. Despite being unimodal, the *d*_h_ distribution is notably broader than those of the other five terpolymers, as manifested by the higher PDI ranging from 0.20 at 45 °C to 0.16 at 50 °C (Figure S8). Upon reaching the onset temperature of the second transition stage at 51 °C, the *Z*-average *d*_h_ first slightly increases to about 270 nm, then gradually decreases to about 180 nm with further heating to 55 °C (Figure 3b). A reduction in larger species is also noticed, as the upper boundary of the *d*_h_ distribution extends to approximately 800 nm at 45–50 °C but gradually decreases to around 300 nm at 51–55 °C (Figure S7). Simultaneously, the PDI decreases to 0.06–0.03 during this period (Figure S8). These results might indicate that an additional morphological transition may occur in the aggregates of OPD(45-35-20) prior to its second phase transition, leading to the formation of a smaller and more uniform species.

The derived count rate (DCR) from the DLS tests, shown in Figure S8, serves as an indicator of scattered light intensity.^57,58^ As shown in Figure S8, the DCR vs temperature profiles of the six terpolymers typically exhibit a steep decline near the corresponding *T*_cp_. This can be attributed to the significant decrease in the number concentration of the scattering particles when they start to aggregate.^57,58^ Following the phase transition, a gradual recovery of DCR is observed, likely reflecting the development of the large thermo-induced aggregates that scatter light more intensively.^57,59^

TEM was employed to investigate the aggregate morphology at four representative temperatures of 25, 40, 48, and 55 °C. The obtained micrographs and particle size histograms are presented in Figures S9 and S10, respectively. As shown in Figure S9, the micrographs display a major presence of spherical species, with their size on a comparable magnitude to the number-averaged *d*_h_ determined by DLS. At 25 and 40 °C which are below the *T*_cp_s, the most abundant species are the primary micelles with a diameter of 10–30 nm. Upon further heating above the *T*_cp_ to 48 °C and subsequently to 55 °C, large spherical species extending to several hundred nanometres in diameter are observed. Interestingly, the aggregates of OPD(45-35-20) show a vesicle-like morphology at 48 °C which is within its first stage of phase transition. At 55 °C, despite the more irregular appearance observed in the representative micrograph, further structural determination was hindered by the artifacts introduced by drying effect and the limited number of particles captured. In order to address these limitations and gain deeper but more reliable insights into the aggregation behavior of OPD(45-35-20), VT-^1^H NMR and SAXS were employed to further investigate the aggregation process of OPD(45-35-20) in its solution state and the potential origin of its dual-stage phase transition.

#### 3.3.3 Chain Immobilization

The phase transition on the molecular level was investigated using VT-^1^H NMR as the thermo-induced immobilization of the polymer chain can be evaluated by the attenuation of the corresponding peak.^49−51^ Although this technique is considered as an effective tool to characterize the thermo-induced behaviors of the terpolymers on the molecular level, it should be noted that the thermoresponsive properties in D_2_O may slightly vary from the counterparts in H_2_O due to the isotope effects.^60−62^ In our case, the phase transition behaviors of the terpolymers in D_2_O solution are similar to those in H_2_O solution, as shown in the UV–vis spectrum in Figure S11. However, the *T*_cp_s in D_2_O slightly deviate from those in H_2_O, as listed in Table S1.

**Figure 4:**
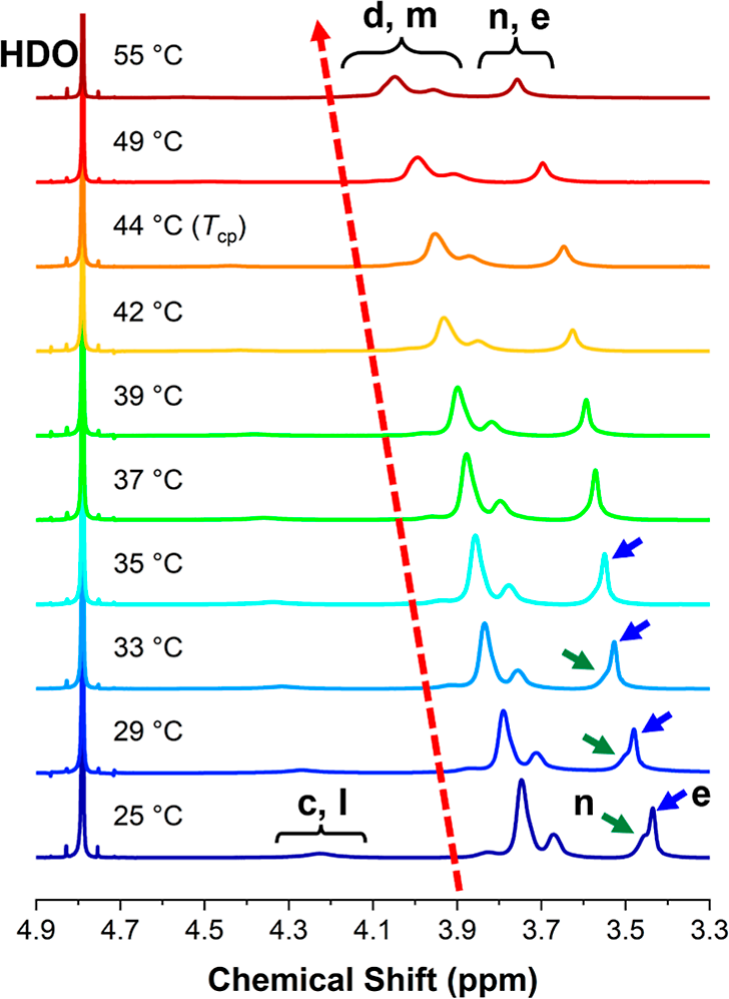
Representative VT-^1^H NMR spectra of OPD(45-25-30) at 1 wt % in D_2_O from 25 to 55 °C.

The representative VT-^1^H NMR spectra of OPD(45-25-30) acquired from 25 to 55 °C are presented in Figure 4 as examples. In this set of spectra, the water residual peak at 4.79 ppm was set as an internal reference for peak positioning. A progressive shifting is noticed in all the polymer peaks toward the lower field (higher ppm) upon heating, marked by the red arrow. This feature can be attributed to the diminishing hydrogen bonds between the polymer and solvent molecules.^52,63,64^ In the broad temperature range from 25 to 37 °C which is well below the *T*_cp_, only a minor extent of attenuation can be noticed for the peak of the OCH_2_CH_2_ group (*d*, *m*). This feature is likely due to the strong hydrophilicity of the OCH_2_CH_2_ group, which can sustain the interactions with water and hence the mobility. Interestingly, compared to the ^1^H NMR spectrum at 25 °C, the characteristic peak of poly(DEGMA), the peak *n* for the terminal OCH_3_ group (marked by green arrows), becomes broadened and less pronounced in the range of 29–35 °C, whereas the counterpart of poly(OEGMA300), marked by blue arrows, remains almost unchanged. This feature may indicate a sequential immobilization of the poly(DEGMA) block followed by the poly(OEGMA300) blocks. It was reported that the *T*_cp_ of poly(DEGMA) homopolymer is about 27–30 °C which is greatly lower than that of poly(OEGMA300).^36^ Therefore, the mobility of the poly(DEGMA) block in the terpolymer can be severely reduced in the relevant temperature zone, prior to any pronounced immobilization of the poly(OEGMA300) block. When the temperature approaches the *T*_cp_ (>39 °C for OPD(45-25-30)), all the polymer peaks start to attenuate and broaden, which can be attributed to the immobilization of the poly(OEGMA300) block. Above the *T*_cp_, only a weak signal persists, likely due to the residual bound solvent.^50^

To quantitatively evaluate the thermo-induced immobilization of the terpolymers on the molecular level, an immobilization factor, *p*, was employed to characterize the temperature-dependent immobilization of the poly(OEGMA300) and poly(DEGMA) blocks.^50,51,65^ The value of *p* was calculated by
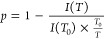
2

In this equation, *I*(*T*) is the integral intensity of each peak at a given temperature *T*, while *I*(*T*_0_) denotes the integral intensity of the peak at the reference temperature *T*_0_, where the maximum mobility is observed (set to 25 °C), and thus the *p*-value here should be zero. The factor *T*_0_/*T* serves as a calibration coefficient, accounting for the intrinsic attenuation that occurs with the increasing temperature.^66^

**Figure 5:**
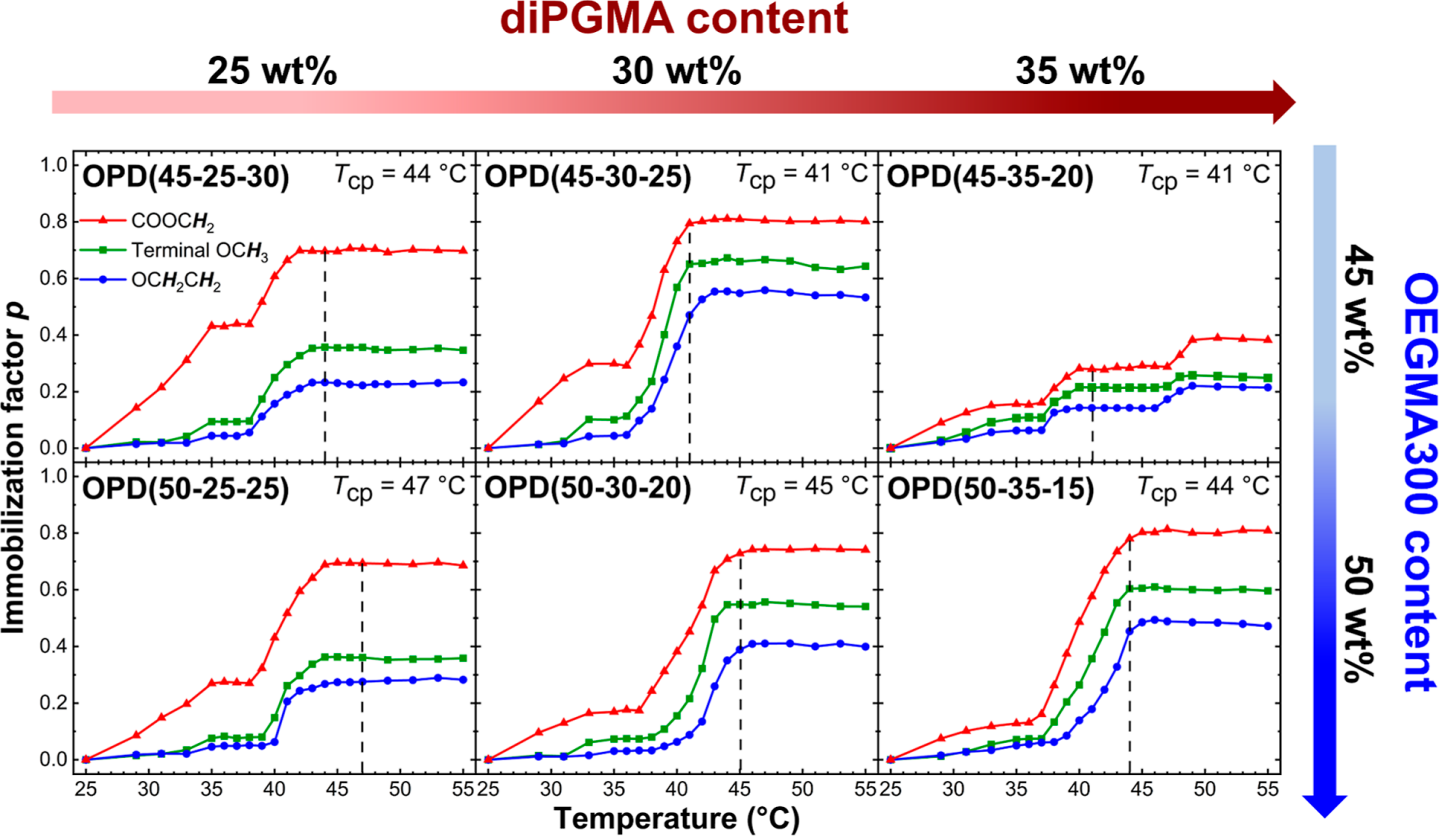
Temperature dependence of the immobilization factor *p* of the six terpolymers. The dashed line indicates the corresponding *T*_cp_ determined by UV–vis in 1 wt % D_2_O solution.

The COOCH_2_, OCH_2_CH_2_, and terminal OCH_3_ groups from the side chains of the poly(OEGMA300) and poly(DEGMA) blocks are selected to characterize the thermo-induced immobilization of the polymer chains. The temperature-dependence of the immobilization factor, *p*, for the three chemical groups from the six terpolymers is shown in Figure 5. Generally, the three groups exhibit varying sensitivities to temperature. Judging from the magnitude of the *p*-values, the COOCH_2_ group is clearly the most sensitive one against the temperature change, followed by the terminal OCH_3_, and OCH_2_CH_2_ at last. This trend aligns well with the hydrophilicity sequence of the groups since the group with higher hydrophilicity should hence possess a stronger resistance to the thermo-induced dehydration and immobilization.

The *p*-values of the five terpolymers displaying a sharp phase transition exhibit a similar evolving trend to each other. From 25 °C to about 37 °C, the *p*-values of the three groups gradually increase from zero to an initial plateau. This feature corresponds well to the sequential immobilization of the poly(DEGMA) block followed by the poly(OEGMA300) block. More specifically, the immobilization of the poly(DEGMA) block is nearly completed at about 35–37 °C, while that of poly(OEGMA300) has barely started. This feature also indicates that the hydrophilicity of the poly(OEGMA300) block is sufficient to stabilize the micellar structure and prevent aggregation, despite the dehydration and immobilization of the poly(DEGMA) block.

Above 37 °C, the *p*-values increase again and reach the second plateau in a higher magnitude, indicating the completion of the immobilization of the poly(OEGMA300) block. The emergence of the second plateau around the *T*_cp_ may suggest that the macroscopic phase transition is predominantly governed by the immobilization of the poly(OEGMA300) block. This progressive evolution of the *p*-values also evident that the thermo-induced immobilization is a continuous process commencing from an earlier stage, rather than a sudden event occurring at the *T*_cp_. This feature is quite similar to the micellar aggregation determined by DLS (vide supra).

Unlike the other five terpolymers, OPD(45-35-20) exhibits a tristage evolution of the *p*-values, aligning well with its dual-stage phase transition on the macroscopic level. From 25 °C to its *T*_cp_, the evolving trend is similar to the other five terpolymers, with the *p*-values gradually increasing to the first plateau due to the immobilization of poly(DEGMA) block, and further reaching the second plateau at a slightly higher magnitude upon heating to the *T*_cp_ at 41 °C. In contrast to the other five terpolymers that cease from further immobilization above the respective *T*_cp_, the *p*-values of OPD(45-35-20) continue to increase from about 47 °C until reaching a third plateau at 49 °C, which is approximately the onset temperature of its second phase transition, as determined in the UV–vis spectrum in Figure S11. Notably, the plateaued *p*-values of the latter two stages are significantly lower than expected, suggesting a possible rearrangement of the poly(OEGMA300) block onto the interface between the polymer-rich domain and the water-rich domain, thus improving the chain hydration and mobility.

#### 3.3.4 SAXS Analysis

**Figure 6:**
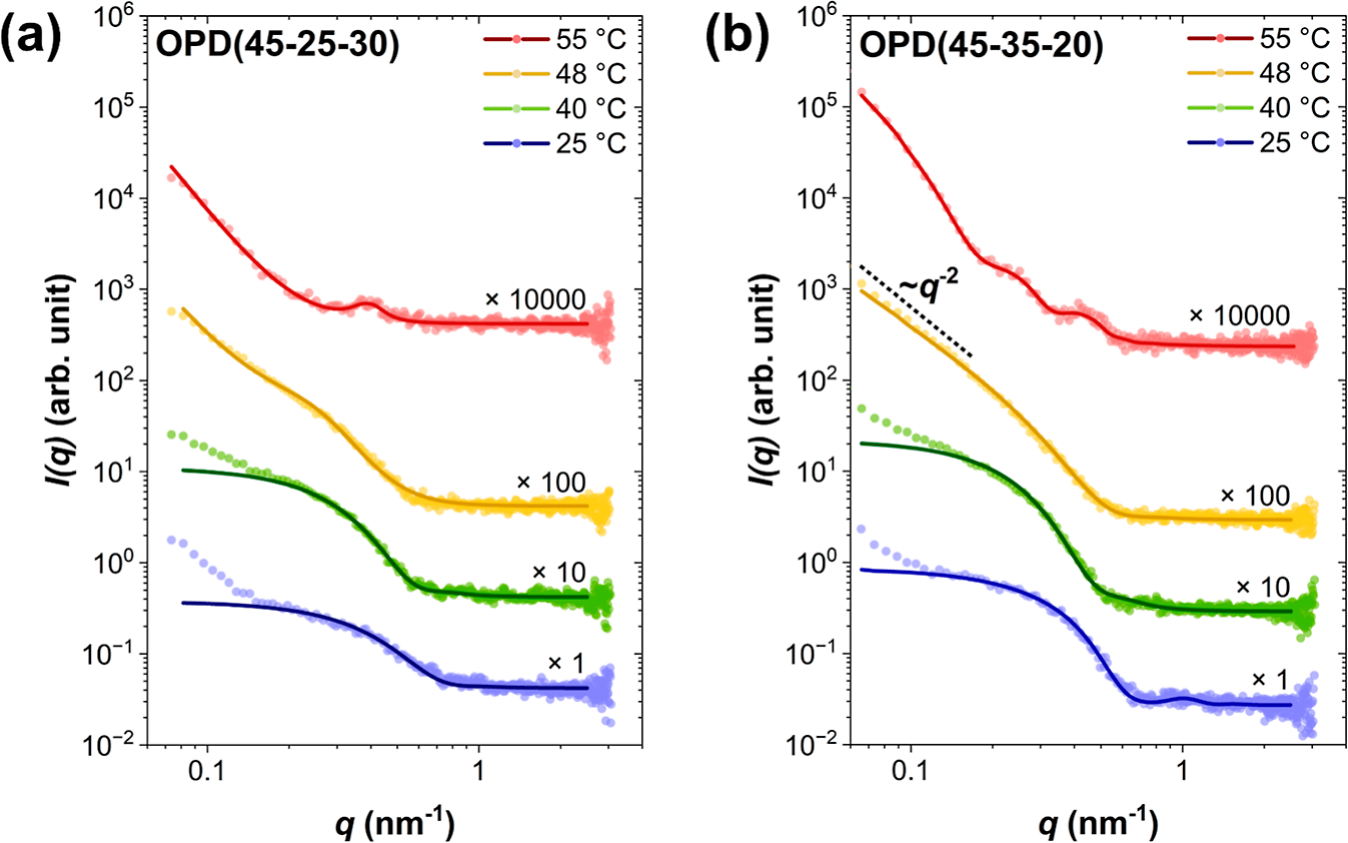
SAXS profiles of (a) OPD(45-25-30) and (b) OPD(45-35-20) at 1 wt % in DI water at 25 °C (blue), 40 °C (green), 48 °C (yellow), and 55 °C (red). The dots represent the experimental data while the lines are for the modeled results. For clarity, the curves are shifted by multiplying a scaling factor.

As determined by UV–vis, DLS, TEM, and VT-^1^H NMR, the five terpolymers displaying a rapid phase transition demonstrate similar properties to each other on both macroscopic and microscopic level, despite having different *T*_cp_s. However, OPD(45-35-20), which shows a dual-stage transition, behaves more distinctively. Therefore, SAXS was employed to reveal further details on the aggregate structures in the polymer solutions. For this purpose, OPD(45-25-30) was selected as a representative for the five rapid-transitioning terpolymers as its transition processes take place in comparable temperature ranges to those of OPD(45-35-20). The SAXS profiles of these two terpolymers, collected at the four representative temperatures, i.e., 25, 40, 48, and 55 °C, are presented in Figure 6.

At 25 and 40 °C, both OPD(45-25-30) and OPD(45-35-20) exhibit SAXS profiles characterized by a plateau in the scattering vector (*q*) range of 0.15–0.3 nm^–1^, followed by a strong decay in the intermediate *q* range of 0.3–0.8 nm^–1^. This pattern suggests that isolated spherical micelles are likely the predominant species present.^67,68^ Due to the limited contrast in the scattering length density (SLD) between the micelle core, shell, and solvent, the experimental SAXS profiles were only modeled using the simple sphere model (eq S1). The derived structural parameters, detailed in Table S2, indicate the radii of the micelles for OPD(45-25-30) and OPD(45-35-20) are approximately 5 and 6 nm at 25 °C, which slightly increase to 6 and 8 nm at 40 °C, respectively. However, a mild upturn in the low *q* range (<0.15 nm^–1^) deviates significantly from the model fits, suggesting the presence of large clusters even below the *T*_cp_. This observation aligns with findings reported for other thermoresponsive polymers.^69−73^

At 48 °C for OPD(45-25-30), the plateau characteristic of the isolated spheres is substituted by a more pronounced “tail”, indicative of large clusters, with a weak hump noticed at *q* = 0.26 nm^–1^, representative of the intermolecular interactions.^74^ Given that the phase transition and the formation of large aggregates have already occurred, the spherical micelles are less likely to exist isolated from each other. However, the absence of a distinct correlation peak renders it neither likely to reach a satisfactory fit by coupling the sphere model with a hardsphere or a sticky hardsphere structure factor, which can describe the interactions among the collapsed micelles in micellar aggregates.^75^ Therefore, the residual spheric features from collapsed micelles in the aggregates cannot be resolved from the SAXS profile, and thus it would be inappropriate to analyze the profile with any sphere-based models.

In this scenario, a correlation-length model was employed, comprising a power-law term for the aggregates scattering in the low *q* range and a Lorentz term for the polymer chain scattering in the high *q* range (eq S2).^76^ The structural parameters derived from the model are listed in Table S3. The correlation length, ξ, representing the characteristic distance among the polymer chains or the “mesh size” of the chain network,^74^ is determined to be 3.7 nm, suggesting a dense packing of the polymer chains within the globular aggregates. The Lorentz exponent, *m*, which describes the chain dynamics, is found at 5.35, indicating a low extent of hydrogen bonding between water molecules and the polymer chains at this high temperature.^74^

At 48 °C for OPD(45-35-20), the SAXS profile exhibits a power-law decay in the low *q* range with a slope of approximately −2, characteristic of lamellar structures.^77,78^ The absence of interference peaks suggests that these lamellar objects may exhibit low structural periodicity (probably without stacking) and high dimensional dispersity. Indeed, a closer fit was achieved using the single-layer lamellar model (eqs S3 and S4) over the correlation-length model, yielding a lamella thickness of 9.9 nm with a high PDI of 0.23 (Table S4). Given that the overall size of the scattering objects (*Z*-average *d*_h_ ≈ 230 nm by DLS, Figure 3b) greatly exceeds the detection limit of SAXS, it is likely that this lamellar feature represents a fraction of a larger structure, possibly large vesicles.^78^ Moreover, the inference of vesicle formation is supported by both the vesicular structures observed in TEM (Figure S9) and the lower *p*-values determined from the VT-^1^H NMR spectra (Figure 5). Instead of being embedded and restricted inside the aggregates, the poly(OEGMA300) segments of OPD(45-35-20) could be repositioned at the polymer–water interface of the thin vesicle membrane, resulting in a lower degree of dehydration and immobilization, as derived from the VT-^1^H NMR spectra. Therefore, the micelles of OPD(45-35-20) may initially reassemble into vesicles above its *T*_cp_, rather than directly collapsing and agglomerating into large globular aggregates, as observed in OPD(45-25-30). This distinct transition could be attributed to the appropriate chemical composition of OPD(45-25-30). Specifically, the balanced ratio of its hydrophilic and hydrophobic contents likely leads to a packing structure favoring the micelle-to-vesicle transition upon heating, rather than simple aggregation.

At 55 °C for OPD(45-25-30), the weak “hump” observed at 48 °C becomes more pronounced and shifts to a higher *q* range. To analyze this feature, the broad-peak model was used, which is an empirical combination of a broad Lorentzian peak function and a power-law decay, to describe the correlation between scattering inhomogeneities (eq S5).^79^ The peak position, *q*_0_, is found at 0.39 nm^–1^ (Table S5), corresponding to an interdomain spacing *d*_0_ (*d*_0_ = 2π/*q*_0_) of 16.1 nm which represents the center-to-center distance between the inhomogeneity domains.^80^ The screening length, ξ, derived from the model, is 16.2 nm, which characterizes the dimension of the inhomogeneity domains.^81^ Notably, the proximity of *d*_0_ and ξ suggests that the polymer-rich domains may maintain a compact arrangement within the aggregate network. The emergence of the domain features could be related to a further structural rearrangement within the aggregates during the characterization time frame of SAXS. However, this variation does not appear to have a notable impact on other aggregate properties, as inferred from the results of the complementary techniques.

At 55 °C for OPD(45-35-20), the SAXS profile shows two additional peaks compared to that at 48 °C. Using the former single-layer lamellar model (eqs S3 and S4), the peak intensities and the upturn in the low *q* range were mildly underestimated compared to the experimental results. However, despite this discrepancy, other features, especially the positions of the peaks, can still be fitted well, suggesting the retention of a lamella-like structure. Interestingly, a closer fit was achieved with a combined model of a power-law term and the single-layer lamellar term (eq S6), yielding a power-law exponent, *n,* of 2.8 and an increased membrane thickness of 36.2 nm (Table S6). Compared to the thinner membrane observed at 48 °C, it is expected that more poly(OEGMA300) segments are embedded within the thicker membrane at 55 °C. Consequently, the extent of chain immobilization should be enhanced, which is characterized well by the increased *p-*values derived from the VT-^1^H NMR spectra (Figure 5).

The contribution from an additional power-law term in the low *q* range is often attributed to interfacial scattering of large objects (clusters, aggregates, etc.) that exceeds the probing length of SAXS, reflecting their local structural features.^69,75,76,82−84^ The value of the power-law exponent *n* can be used to evaluate surface roughness of the scattering objects. For example, a smooth surface typically corresponds to *n* ≈ 4. In our case, the *n* value of 2.8 likely indicates a rough surface.^75,83,84^ It could be more reasonable to attribute this scattering feature to the vesicle surface rather than to rough-surfaced aggregate species composed of collapsed vesicles, as this significant structural variation would eliminate the observed scattering features of single-layer lamellae. The coexistence of vesicles and rough aggregates is also ruled out since the narrow *d*_h_ distribution observed via DLS cannot support the presence of a second free-diffusing species (vide supra). Thus, the combination of the single-layer lamellar model and a power-law term could suggest that the observed scattering arises from an altered vesicular species characterized by a rough surface.

Furthermore, it is well-known that the physics of self-assembly imposes a well-defined control over the membrane thickness of polymeric vesicles (polymersomes), typically governed by the degree of polymerization (or chain length) and composition of the polymer building blocks.^85,86^ However, the vesicular species observed at 55 °C exhibits an atypical membrane thickness of 36.2 nm, which is approximately four times the extended chain length of the corresponding polymer (8.6 nm). This substantial discrepancy suggests that it is not possible for the vesicular species to maintain a well-defined membrane structure through the conventional self-assembly approaches of individual polymer chains; instead, it could involve the aggregation of polymer entities in a less ordered arrangement. If this assumption holds, the potential aggregation process that leads to the membrane thickening should also result in a more irregular surface texture, i.e., a roughened surface.

The interpretations gained from the SAXS profiles, combined with the reduction of larger species as well as the decrease in both the *Z*-average *d*_h_ and PDI observed by DLS at this temperature (vide supra), could lead to the following assumption regarding the formation of the scattering objects: vesicles with higher *d*_h_ could be prone to disassembly, while those with lower *d*_h_ are more likely to remain intact. Simultaneously, reaggregation of the resulting polymeric fragments from the disassembled vesicles appears to occur on the surfaces of the remaining ones. During this transition, the disassembly of the large vesicles could contribute to the reduction in overall *d*_h_ and its narrower distribution. Given that the vesicle size significantly exceeds the membrane thickness, the membrane thickening induced by the simultaneous reaggregation should impose a minimal impact on the uniformity of the overall *d*_h_ of the vesicular species. Therefore, the proposed process would ultimately lead to the formation of vesicular objects characterized by a reduced yet more uniform *d*_h_, a thicker membrane, and a roughened surface.

**Figure 7:**
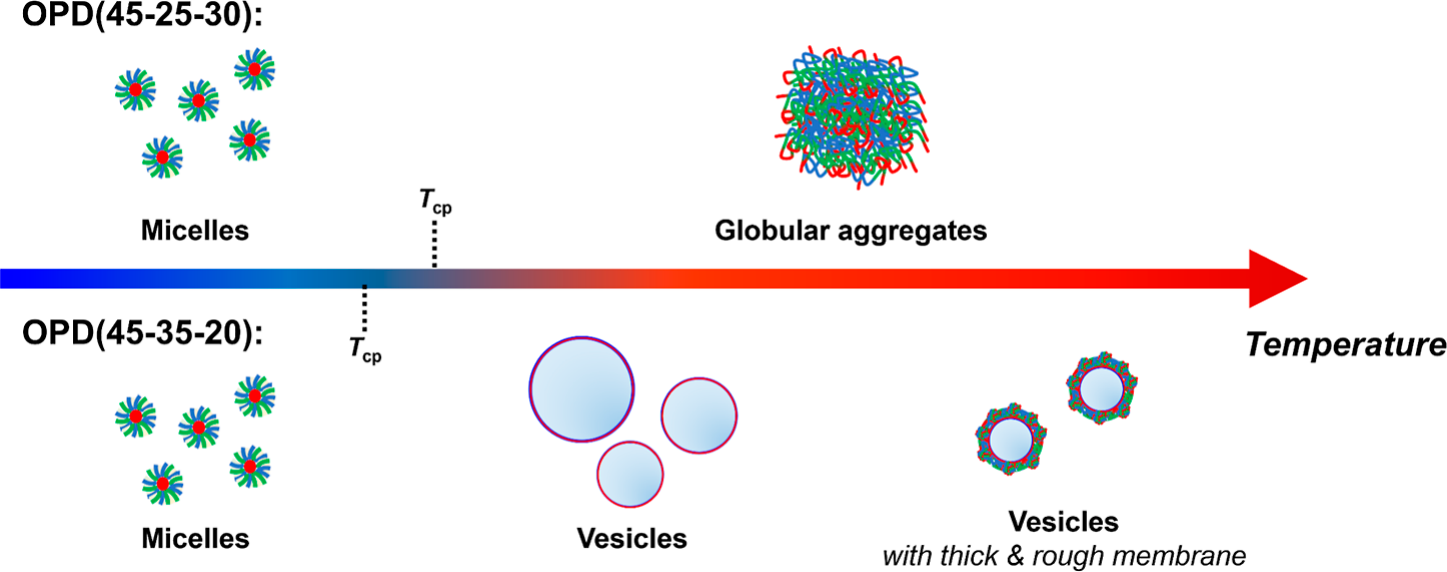
Schematic representation of the thermo-induced aggregation of OPD(45-25-30) and OPD(45-35-20) at 1 wt % in H_2_O upon heating.

Based on the results from SAXS and other complementary techniques, the thermo-induced structural evolutions of OPD(45-25-30) and OPD(45-35-20) are schematically illustrated in Figure 7. At 25 and 40 °C which are below the respective *T*_cp_, the major species in the solutions of both terpolymers are spherical micelles. Despite having the poly(DEGMA) block immobilized from approximately 35–37 °C, the micellar structure is still preserved at 40 °C due to the solvation of the poly(OEGMA300) block.

At 48 °C, both the terpolymers undergo the phase transition due to the immobilization of the poly(OEGMA300) block. For OPD(45-25-30), the extent of chain immobilization reaches the maximum while the micelles collapse and agglomerate into globular aggregates, from which the residual spheric features of the collapsed micelles were unable to be resolved. For OPD(45-35-20) at this temperature, the micelles reorganize into vesicles, in which the chain immobilization is in a notably lower extent than OPD(45-25-30) and other rapid-transitioning terpolymers. Compared with the globular aggregates of OPD(45-35-20), the vesicles formed by OPD(45-35-20) are much smaller (*Z*-average *d*_h_ ≈ 230 nm vs 600 nm).

At 55 °C, features related to domain structures are resolved from the SAXS profile of OPD(45-25-30), which further confirms the compact conformation of the aggregate network. For OPD(45-35-20) which is in its second stage of phase transition, smaller vesicular species exhibiting rougher and thicker surface are found, possibly due to a secondary aggregation. A further chain immobilization is also noticed for OPD(45-35-20) under this condition.

## 4 Conclusion

In this study, six terpolymers based on OEGMA300, diPGMA, and DEGMA with a consistent MM but systematically varied chemical compositions were prepared via one-pot GTP method. The influence of the composition on the thermo-induced phase transition and aggregation behaviors of the terpolymers are investigated in detail via multiple techniques, including UV–vis, DLS, VT-^1^H NMR, and TEM. Specifically, the thermo-induced morphological transition of the two representative terpolymers, OPD(45-25-30) exhibiting a rapid phase transition and OPD(45-35-20) with a dual-stage transition, were determined via SAXS at various temperatures. Compared with OPD(45-25-30) that directly agglomerate into globular aggregates above the *T*_cp_, OPD(45-35-20) can reorganize from spherical micelles to vesicular objects during its unique dual-stage phase transition.

Given that the reported terpolymers are largely composed of biocompatible OEGMA-based monomers and exhibit transition temperatures above body temperature, they could be promising candidates for drug carriers in photothermal therapy, in which induced hyperthermia is commonly involved. Among the terpolymers investigated, the vesicle-forming OPD(45-35-20) may offer greater opportunities to achieve more sophisticated functionalities and enhanced control over the therapeutic performance. Moreover, the insights gained into the aggregation behaviors and structural evolution of these terpolymers in the pre- and post-transition stages will further advance the designing criteria of thermoresponsive block copolymers, facilitating the development of polymeric materials for a wide range of biomedical applications.

## Supporting Information Available

^1^H NMR of diPGMA (Figure S1), the GPC traces of the six studied terpolymers including the precursors (Figure S2), *I*_1_/*I*_3_-concentration plots for CMC determination (Figure S3), illustration of the core–shell model (Figure S4), histograms of *d*_h_ by intensity and by number of the polymeric micelles in 1 wt % aqueous solution at 25 °C (Figures S5 and S6), overall distributions of *d*_h_ by intensity, PDI, and derived count rate of the terpolymers in 1 wt % aqueous solution at various temperatures (Figure S7 and S8), negative-stained TEM micrographs of the terpolymers at 25, 40, 48, and 55 °C (Figure S9), size distribution histograms based on TEM micrographs (Figure S10), transmittance curves of 1 wt % D_2_O solution upon heating (Figure S11), *T*_cp_s in 1 wt % D_2_O solution (Table S1), and detailed information on SAXS models and parameters extracted (eq S1 to S6 and Tables S2 to S6) (DOCX)

## Abbreviations

BuMA: *n*-butyl methacrylate
CMC: critical micelle concentration
CMT: critical micelle temperature
*D̵*: dispersity index
DEGMA: di(ethylene glycol) methyl ether methacrylate
DCR: derived count rate
diPGMA: di(propylene glycol) methyl ether methacrylate
diPGOH: di(propylene glycol) monomethyl ether
DLS: dynamic light scattering
DMAEMA: 2-(dimethylamino)ethyl methacrylate
DPPH: 2,2-diphenyl-1-picrylhydrazyl
EG: ethylene glycol
Et3N: triethylamine
FDA: U.S. Food and Drug Administration
*G*′: storage modulus
GPC: gel permeation chromatography
GTP: group transfer polymerization
LCST: lower critical solution temperature
MEHQ: mequinol
MM: molar mass
Mn: number-average molar mass
MTS: methyl trimethylsilyl dimethylketene acetal
NMR: nuclear magnetic resonance spectroscopy
OEGMA: oligo(ethylene glycol) methyl ether methacrylate
OEGMA300: oligo(ethylene glycol) methyl ether methacrylate with an average molar mass of 300 g/mol
PDI: polydispersity index
PEG: poly(ethylene glycol)
PMMA: poly(methyl methacrylate)
PNIPAM: poly(*N*-isopropylacrylamide)
PPG: poly(propyl glycol)
SAXS: small-angle X-ray scattering
SLD: scattering length density
TBABB: tetrabutyl ammonium bibenzoic acid
Tcp: cloud point temperature
TEM: transmission electron microscopy
Tgel: temperature of gelation
THF: tetrahydrofuran
UV–vis: ultraviolet–visible spectroscopy
VT-1H NMR: variable-temperature proton nuclear magnetic resonance spectroscopy.
