# Supplementary material for: Effect of Composition on the Thermo-Induced Aggregation of Poloxamer-Analogue Triblock Terpolymers
Source: Macromolecules. 2025 Feb 26;58(5):2289–302. doi: 10.1021/acs.macromol.4c02217 (PMC11912521; doi:10.1021/acs.macromol.4c02217)
Supplement: Supplementary file 1 — ma4c02217_si_001.docx [file ma4c02217_si_001.docx]

**Supporting Information**

**Effect of Composition on the Thermo-induced Aggregation of Poloxamer-analogue Triblock Terpolymers**

*Shaobai Wang ^1^, Alberto Alvarez-Fernandez ^2^, Xu Liu ^1^, Sofia Miron-Barroso ^1^, Kelvin Wong ^3^, Stefan Guldin ^3,4^*, Theoni K. Georgiou ^1^**

*^1^ Department of Materials, Royal School of Mines, Imperial College London, SW7 2AZ London, United Kingdom*

*^2^ Centro de Fisica de Materiales (CFM) (CSIC-UPV/EHU), Material Physics Centre, San Sebastian, 20018 Spain*

*^3^ Department of Chemical Engineering, University College London, Torrington Place, WC1E 7JE London, United Kingdom.*

*^4^ Department of Life Science Engineering, Technical University of Munich, 85354 Freising, Germany*

**S1. Molecular and thermoresponsive properties of the terpolymers**

**
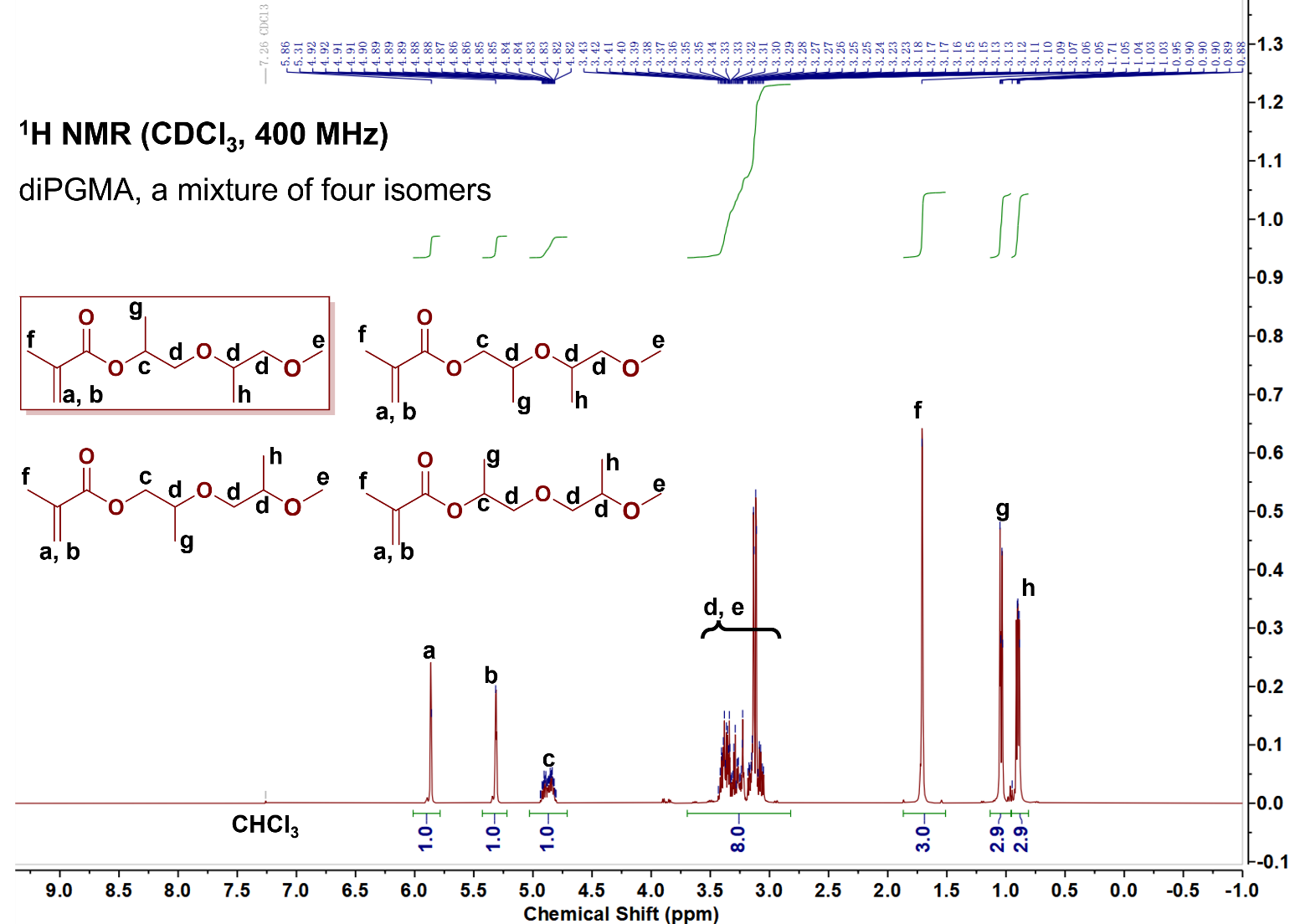
**

**Figure S1.** ^1^H NMR spectrum (400 MHz) of diPGMA in CDCl_3_. The framed structure is for the most abundant isomer.^1^


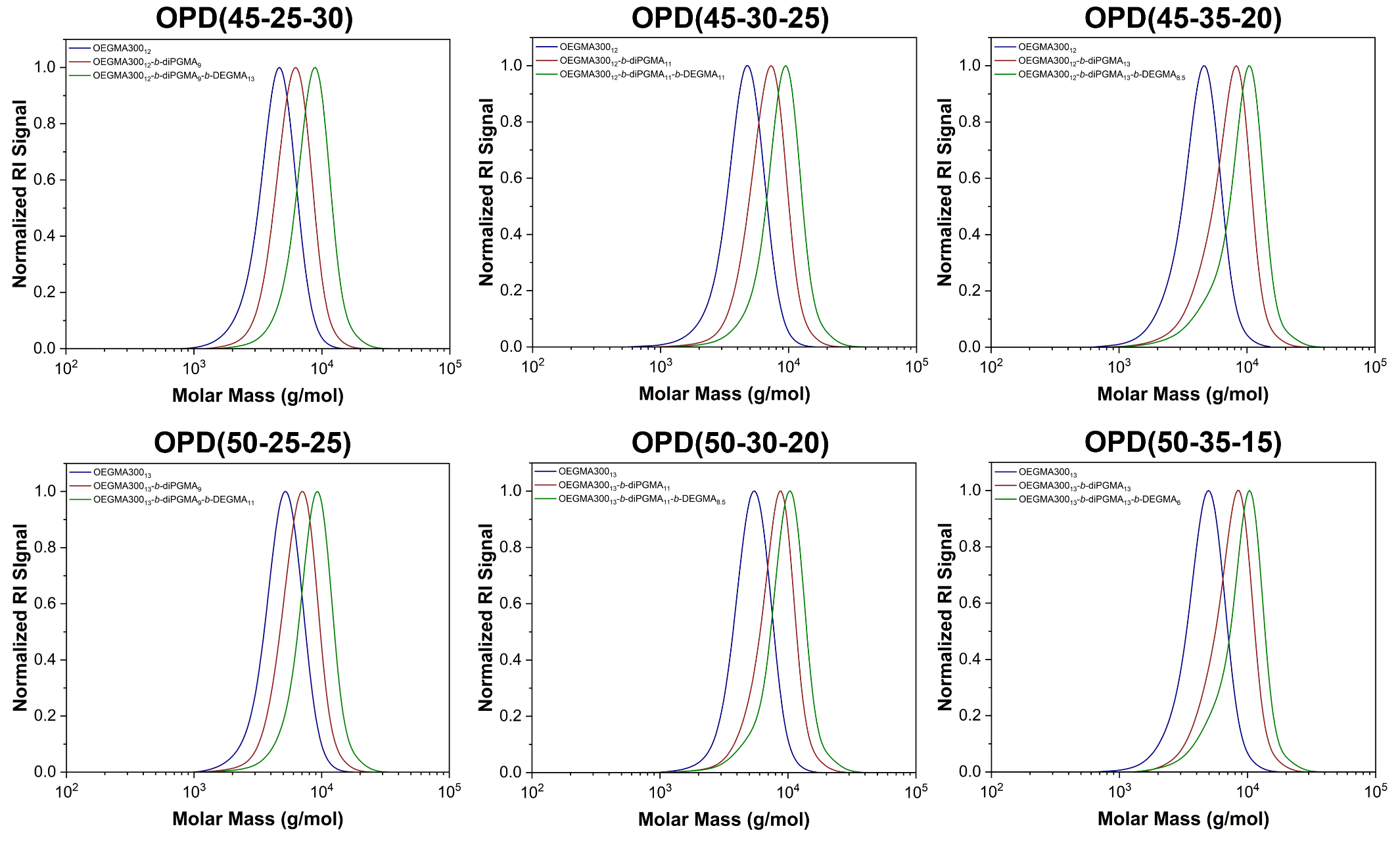


**Figure S2**: The GPC traces of the obtained terpolymers (green) and their corresponding precursors (blue and red) in THF-Et3N (95-5 vol%).


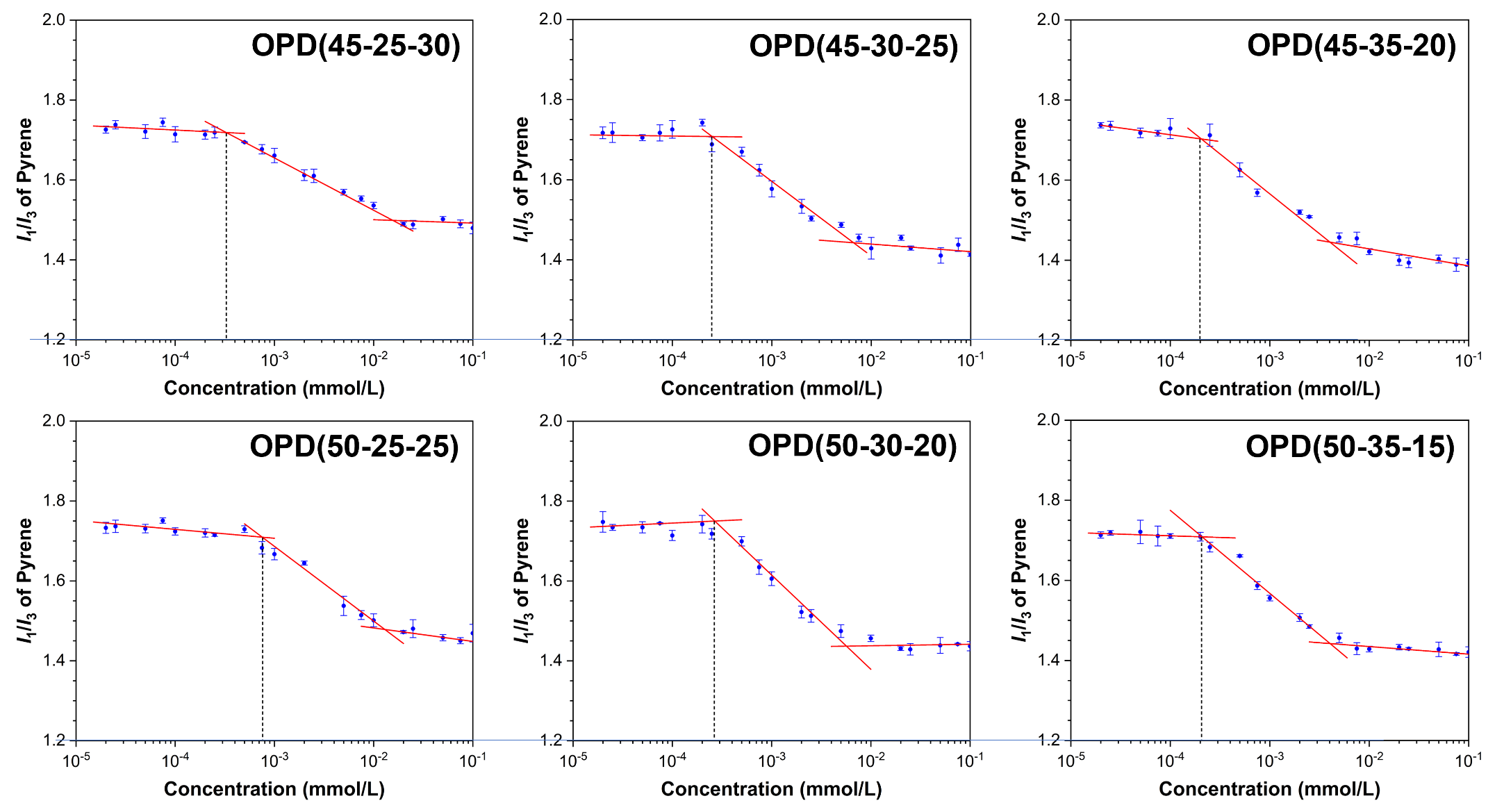


**Figure S3**. The evolution of the intensity ratio, I_1_/I_3_, of pyrene with increasing polymer concentration for CMC determination. The respective CMC is marked with the dotted line.


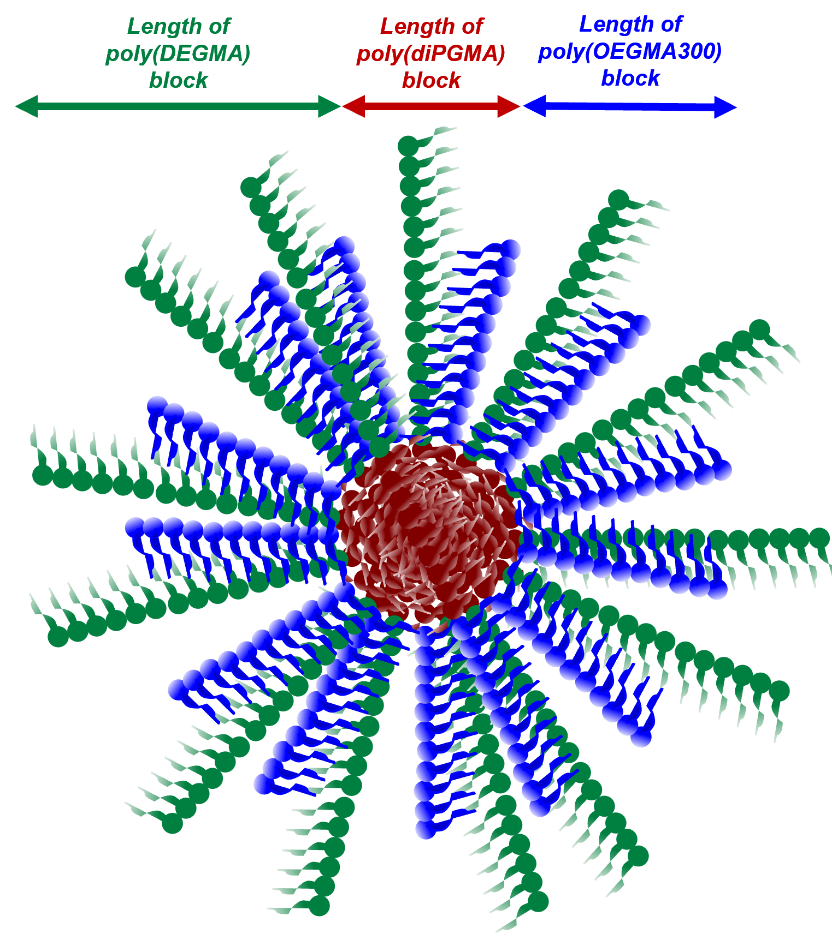


**Figure S4**. The hypothetical conformation of the terpolymer micelles based on the spheric core-shell model, where the hydrophobic poly(diPGMA) core is in red and the hydrophilic corona consisting of poly(OEGMA300) and poly(DEGMA) is in blue and green, respectively. The values of theoretical d_h_ can be calculated by either d_h_ = 0.254 × (DP_diPGMA_ + 2 × DP_OEGMA300_) or d_h_ = 0.254 × (DP_diPGMA_ + 2 × DP_DEGMA_), depending on which hydrophilic block possesses a higher DP. 0.254 (nm) is the projection length of the backbone in a methacrylate repeating unit.


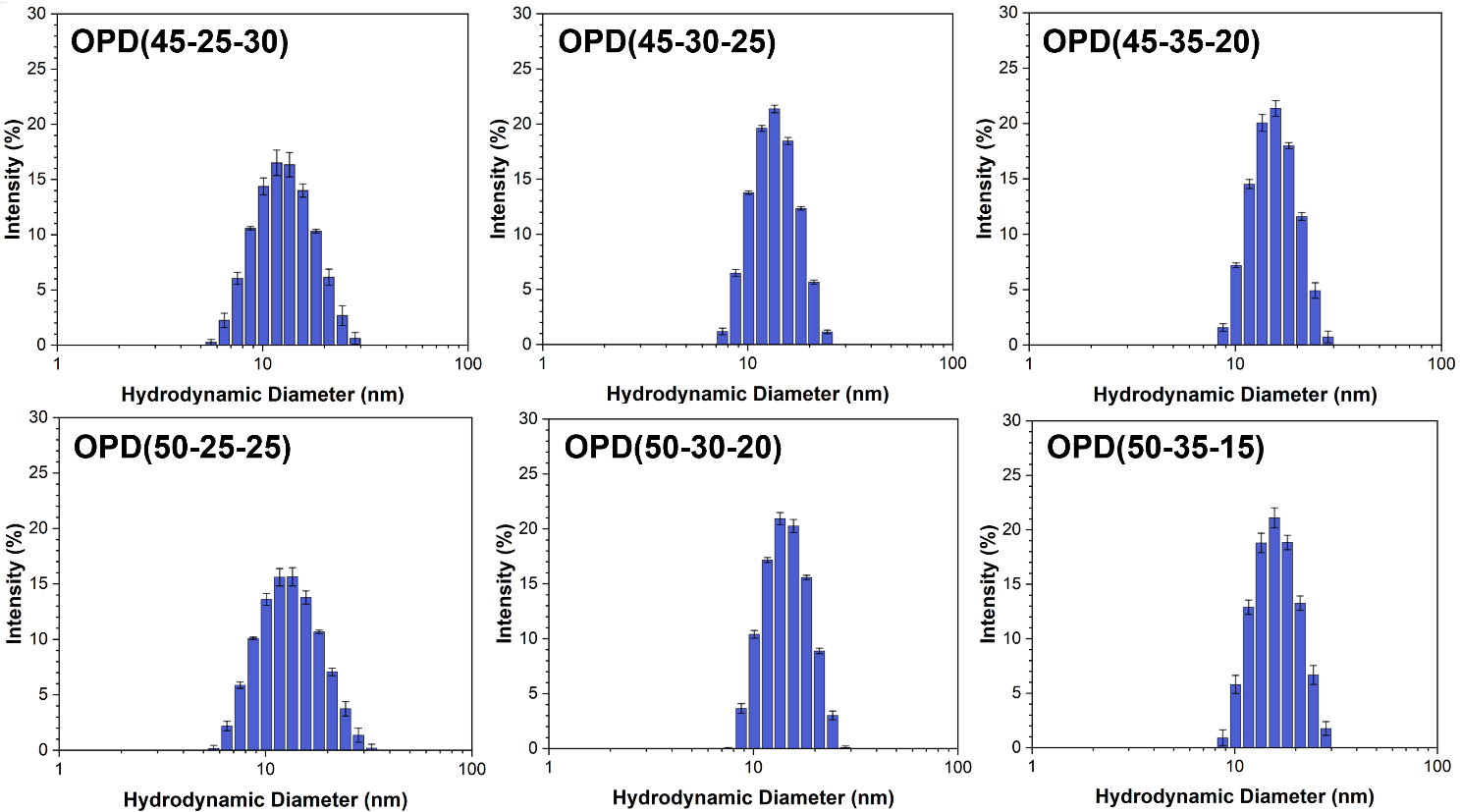


**Figure S5.** Histograms of d_h_ by intensity of the polymeric micelles at 1 wt% in DI water at 25 °C, determined by DLS.


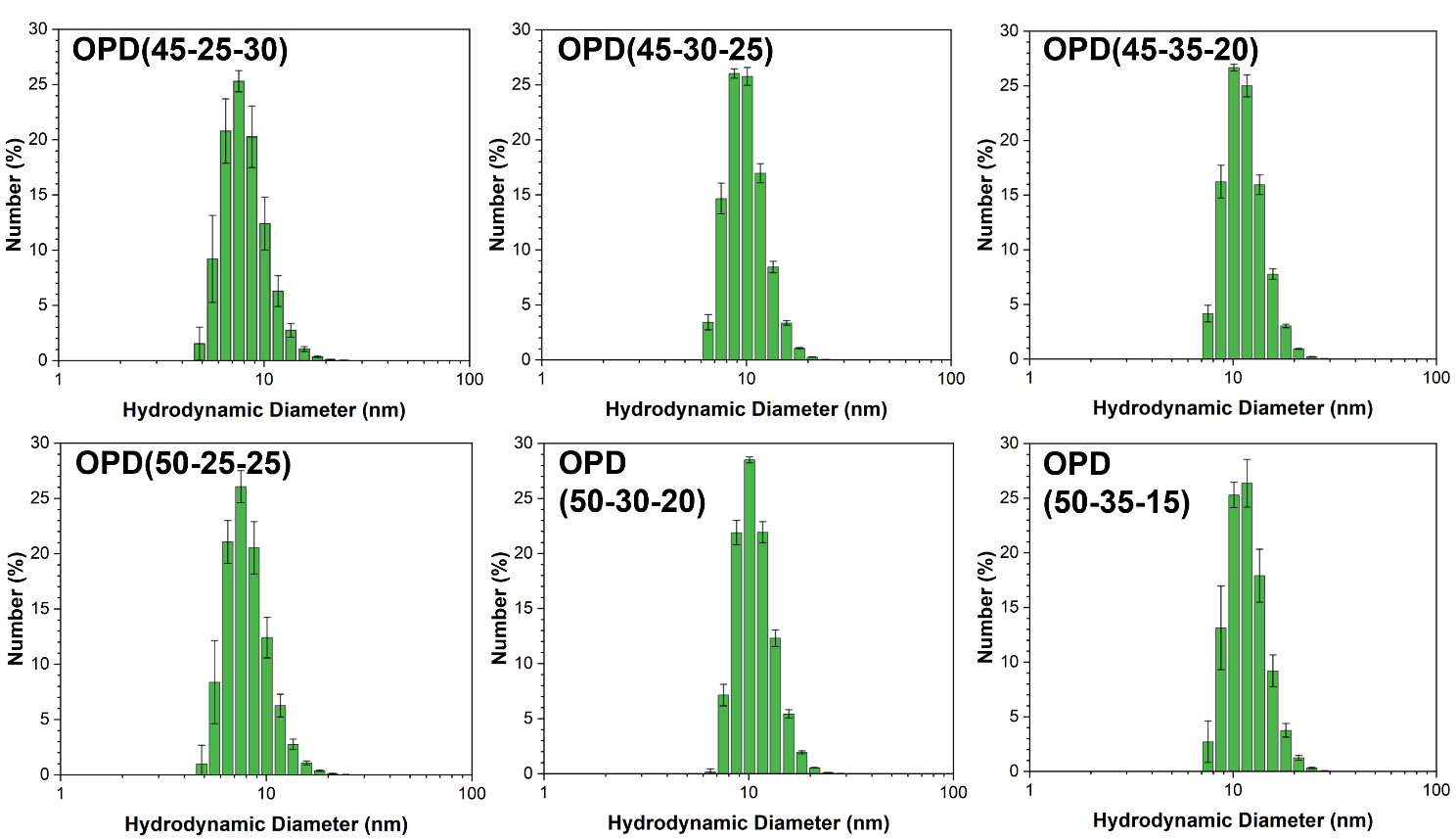


**Figure S6**. Histograms of d_h_ by number of the polymeric micelles at 1 wt% in DI water at 25 °C, determined by DLS.


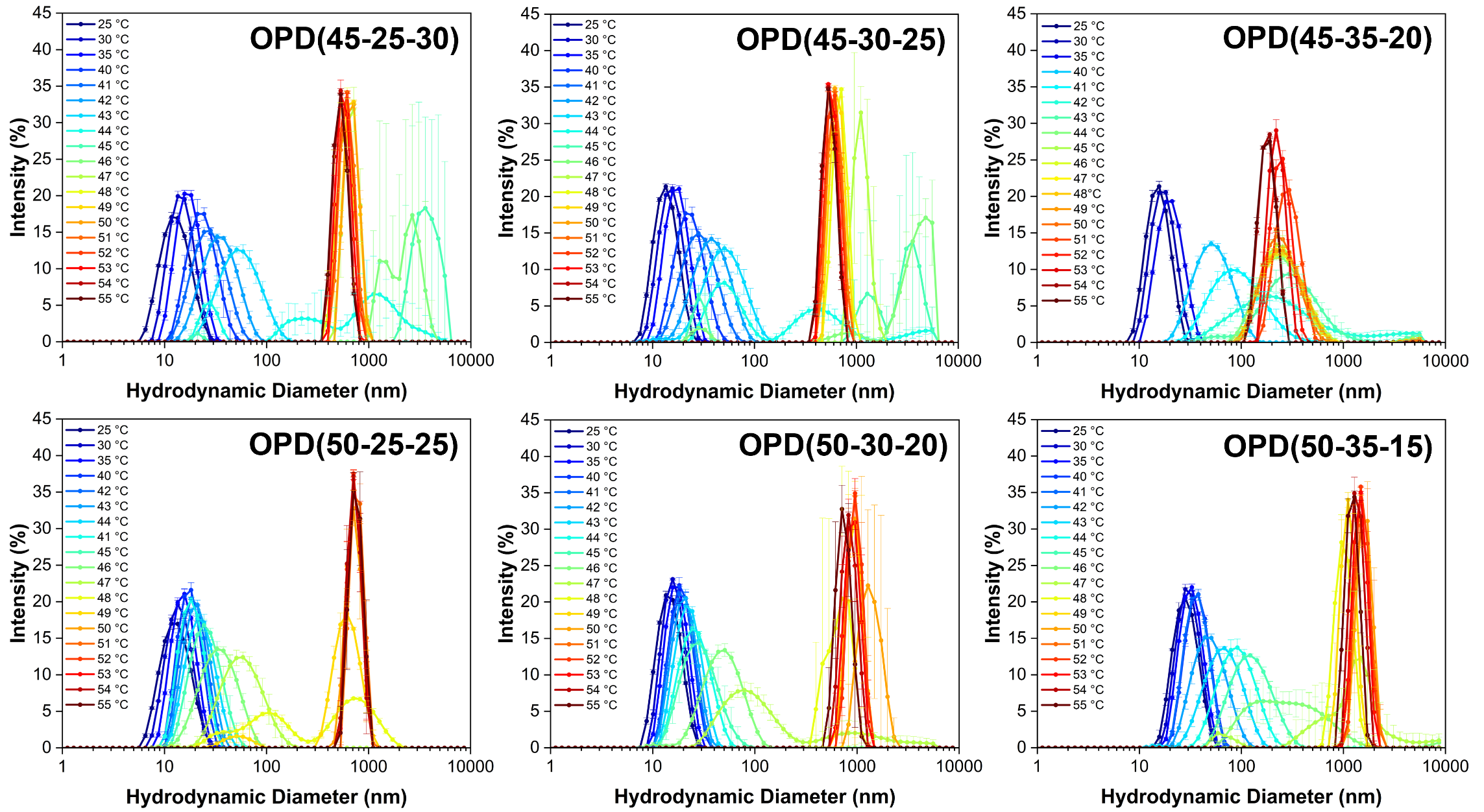


**Figure S7**. Overall distributions of d_h_ by intensity of the polymers in 1 wt% aqueous solution at various temperatures.

**
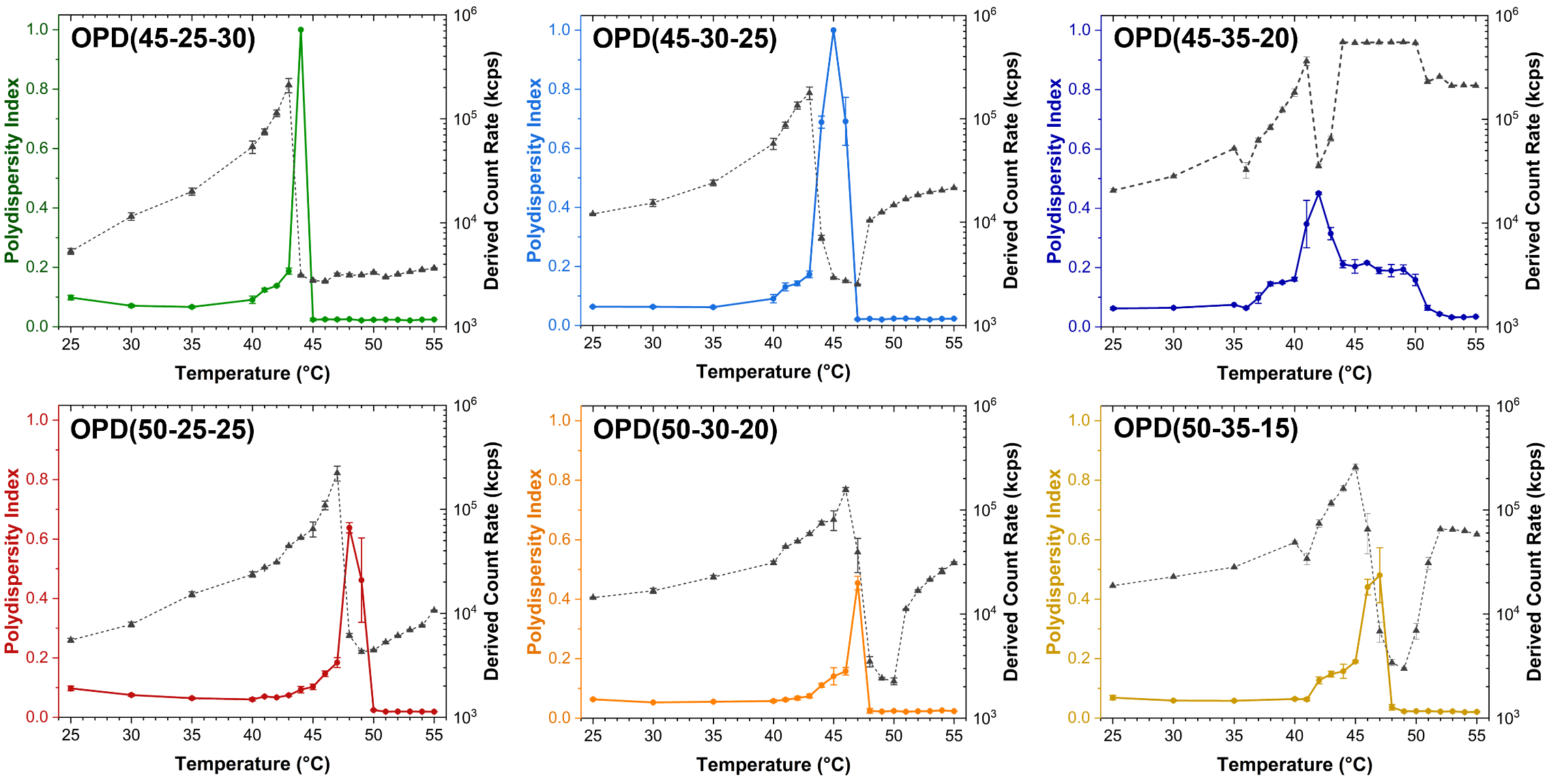
**

**Figure S8.** The polydispersity index (coloured solid curve) and derived count rate (dark dotted curve), as functions of temperature, of intensity-based d_h_ of the terpolymers at 1 wt% in DI water.


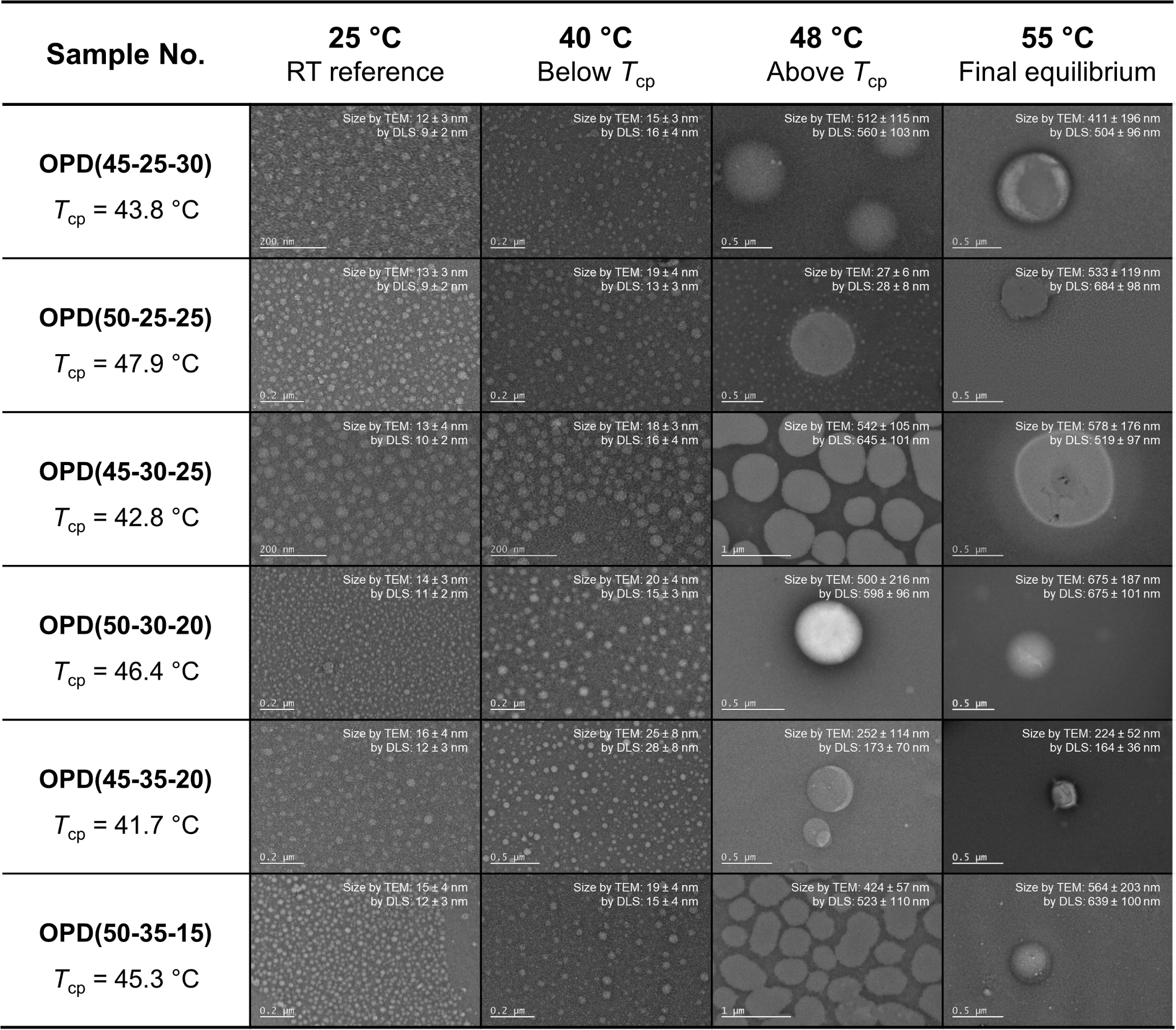


**Figure S9.** Representative TEM micrographs of the micelles/aggregates formed in 1 wt% H_2_O solution at 25, 40, 48, and 55 °C. “Size by DLS” here refers to the number-average *d*_h_ obtained from DLS at the respective temperature.


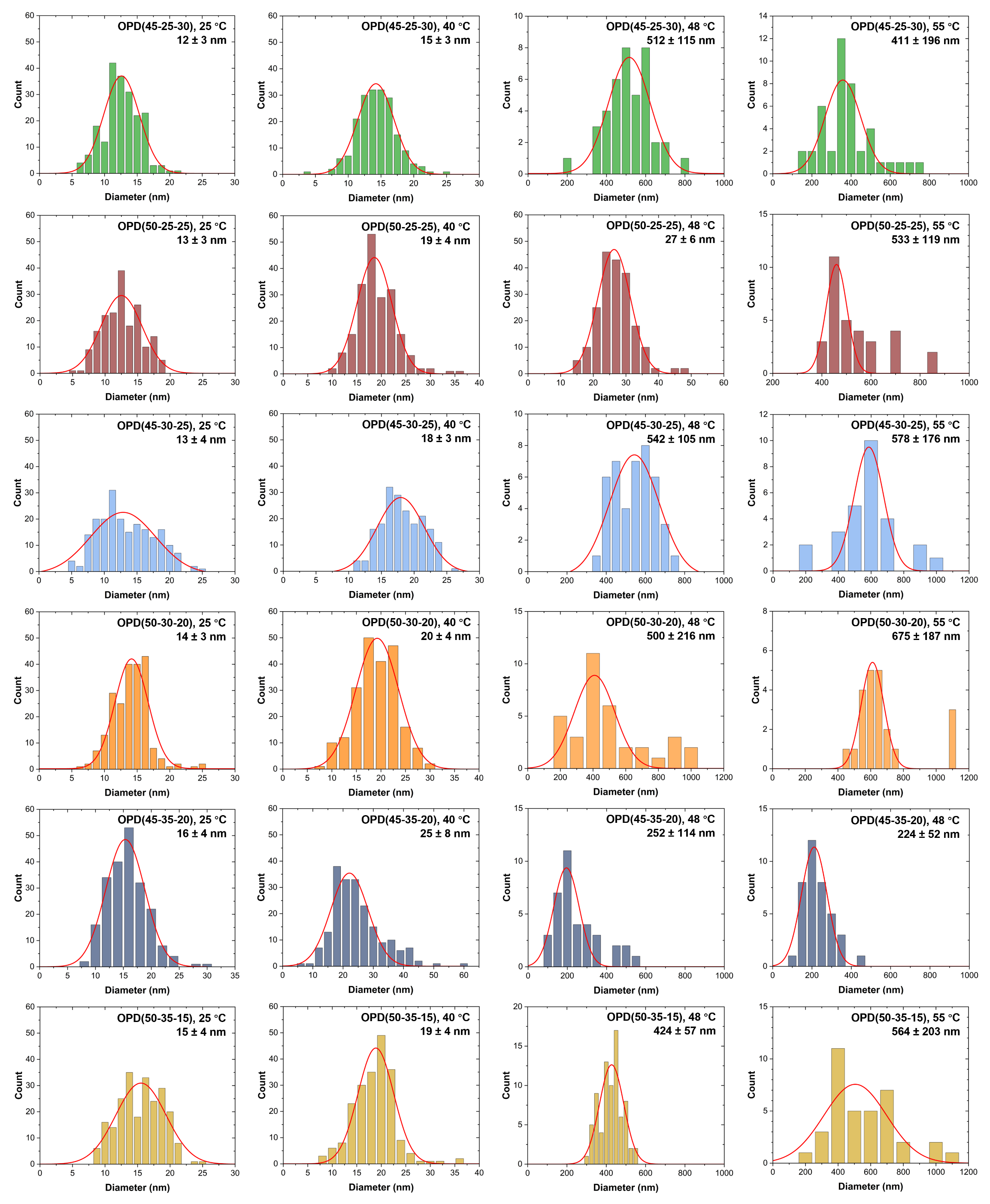


**Figure S10.** Particle size distribution histograms obtained from TEM micrographs.


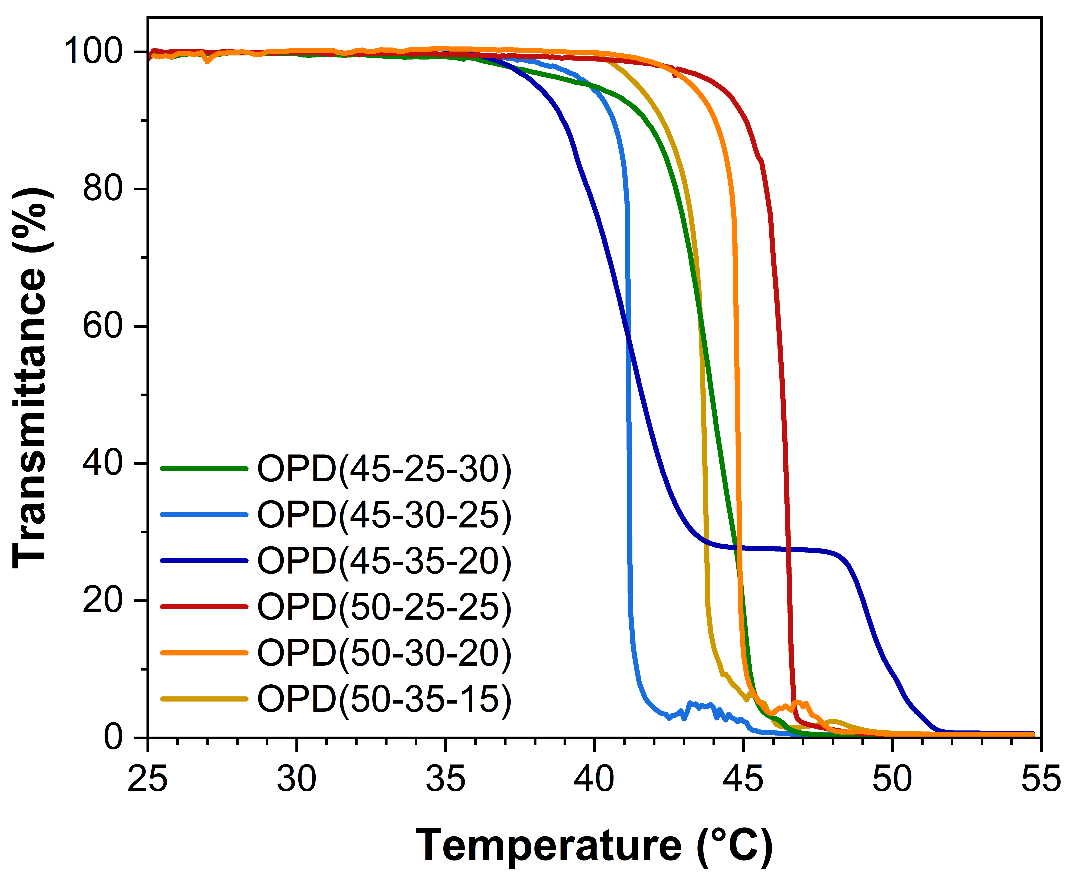


**Figure S11.** Variation of transmittance, as a function of temperature, of the terpolymers at 1 wt% in D_2_O under a testing wavelength of 550 nm and a heating rate of 0.2 °C/min.

**Table S1**. T_cp_ of the terpolymers at 1 wt% in D_2_O solution, determined by UV-Vis with a test wavelength of 550 nm and a heating rate of 0.2 °C.

| **Sample No.**  **(Composition)** | ***T*_cp_ in 1 wt% D_2_O solution**  **(± 0.1 °C)** |
| --- | --- |
| OPD(45-25-30) | 44.0 |
| OPD(45-30-25) | 41.1 |
| OPD(45-35-20) | 41.4 |
| OPD(50-25-25) | 46.5 |
| OPD(50-30-20) | 44.8 |
| OPD(50-35-15) | 43.6 |

**S2. Detailed information on the SAXS fittings**

For the 1 wt% H_2_O solution of OPD(45-25-30) and OPD(45-35-20) at 25 and 40 °C, the experimental SAXS profiles fit well to the simple sphere model. In this model, the scattering intensity is defined as:^2^

|  | $I_{s}\left( q \right)=\frac{I_{0}}{V}\cdot\left[ 3V\left( \Delta\rho\right)\cdot\frac{\sin\left( qr \right)-qrcos\left( qr \right)}{\left( qr \right)^{3}} \right]^{2}+I_{bkg}$ | Eq. S1 |
| --- | --- | --- |

Here, *I*_0_ is a scaling factor that related to the volume fraction of the scatterers. *I*_bkg_ is the background intensity that is fixed at about 0.03 arb. unit. The variables *V* and *r* represent the volume and the radius of the spherical scatter, respectively (*V* = 4*πr*^3^/3). $\Delta\rho$ is the difference of scattering length density (SLD) between the solvent and the scatter, i.e., *Δρ = ρ - ρ*_solv_, where *ρ* is the SLD of the scattering object and *ρ*_solv_ is the SLD of water (9.47 × 10^-6^ Å^-2^). The polydispersity of the radius, *p_r_*, was also obtained from the fitting. The results derived from this model are summarised in Table S2.

**Table S2.** Summary of structural parameters derived from the simple sphere model for OPD(45-25-30) and OPD(45-35-20) in 1 wt% H_2_O solution at 25 and 40 °C.

| **Sample No.** | ***T* (°C)** | ***I*_0_** | ***ρ* (×10^-6^ Å^-2^)** | ***r* (nm)** | ***p_r_*** |
| --- | --- | --- | --- | --- | --- |
| OPD(45-25-30) | 25 | 0.018 ± 0.001 | 10.01 ± 0.04 | 4.6 ± 0.0 | 0.20 ± 0.00 |
|  | 40 | 0.012 ± 0.002 | 10.23 ± 0.07 | 6.2 ± 0.0 | 0.20 ± 0.00 |
| OPD(45-35-20) | 25 | 0.016 ± 0.001 | 10.13 ± 0.04 | 6.2 ± 0.1 | 0.13 ± 0.03 |
|  | 40 | 0.013 ± 0.002 | 10.22 ± 0.07 | 7.6 ± 0.7 | 0.23 ± 0.10 |

For the 1 wt% H_2_O solution of OPD(45-25-30) at 48 °C, the obtained result fits well with the correlation-length model. In this model, the scattering intensity is defined as:

|  | $I\left( q \right)=\frac{A}{q^{n}}+\frac{C}{1+{(q\xi)}^{m}}+I_{bkg}$ | Eq. S2 |
| --- | --- | --- |

The first power-law term here is to describe the scattering feature from the clusters, while the second term is to characterise the polymer/solvent interactions.^3^ The variables *A* and *C* are multiplicative factors and the exponents *n* and *m* are the power-law and Lorentz exponents, respectively. Finally, *ξ* is the correlation length for the polymer chains. The derived results are listed in Table S3.

**Table S3**. Structure parameters derived from the correlation-length model for the 1 wt% H_2_O solution of OPD(45-25-30) at 48 °C.

| **Sample Νο.** | ***T* (°C)** | ***A*** | ***n*** | ***C*** | ***ξ* (nm)** | ***m*** |
| --- | --- | --- | --- | --- | --- | --- |
| OPD(45-25-30) | 48 | (4.0 ± 0.5) × 10^-7^ | 3.42 ± 0.04 | 0.56 ± 0.06 | 3.7 ± 0.1 | 5.35 ± 0.14 |

In the case of OPD(45-35-20) at 48 °C, SAXS data was fitted using a lamellar model. The selection of this model is based on the size of the obtained structures determined by DLS, which is far above the detection limit of SAXS (*d* ≈ 100 nm). This lamellar structure may share similarities to the membrane of a vesicle in a SAXS characterization.

The scattering intensity *I_l_(q)* in the lamellar model can be defined by:

|  | $I_{l}\left( q \right)=\frac{2\pi\varphi P_{l}\left( q \right)}{q^{2}\delta}+I_{bkg}$ | Eq. S3 |
| --- | --- | --- |

and the form factor *P_l_(q)* is:

|  | $P_{l}\left( q \right)= \frac{4({\rho-\rho_{solv})}^{2}}{q^{2}}\sin^{2}\left( \frac{q\delta}{2} \right)$ | Eq. S4 |
| --- | --- | --- |

where *δ* is the total layer thickness, while *ρ* and *ρ*_solv_ are the SLDs of the fitted species and the solvent (9.47 × 10^-6^ Å^-2^), respectively.^4^ During the fitting process, the polydispersity of the lamellar thickness, *p_δ_*, was acquired from the gaussian distribution of *δ*. The obtained results are listed in Table S4.

**Table S4.** Structural parameters derived from the lamellar model for 1 wt% OPD(45-35-20) in H_2_O at 48 °C.

| **Sample No.** | ***T* (°C)** | ***φ*** | ***ρ* (×10^-6^ Å^-2^)** | ***δ* (nm)** | ***p_δ_*** |
| --- | --- | --- | --- | --- | --- |
| OPD(45-35-20) | 48 | 0.008 ± 0.001 | 10.37 ± 0.08 | 9.9 ± 0.2 | 0.23 ± 0.07 |

For the 1 wt% solutions of OPD(45-25-30) at 55 °C, the data fits well with the broad-peak model, which is described by:

|  | $I\left( q \right)=\frac{A}{q^{n}}+\frac{C}{1+{(\left\vert q-q_{0} \right\vert\xi)}^{m}}+I_{bkg}$ | Eq. S5 |
| --- | --- | --- |

The first term is the power-law scattering factor that describes the scattering from clusters. The second term is related to the inhomogeneities of the system, where *C* is the Lorentz scaling factor, *q*_0_ is the peak position that is related to the characteristic distance between the scattering inhomogeneities, *ξ* is the screening length, and *m* is the Lorentz exponent. The results are listed in Table S5.

**Table S5.** Structural parameters derived from the broad-peak model for 1 wt% OPD(45-25-30) in H_2_O at 55 °C.

| **Sample Νο.** | ***T* (°C)** | ***A*** | ***n*** | ***C*** | ***q_0_* (Å^-1^)** | ***ξ* (nm)** | ***m*** |
| --- | --- | --- | --- | --- | --- | --- | --- |
| OPD(45-25-30) | 55 | (2.9 ± 0.2) × 10^-8^ | 3.69 ± 0.01 | 0.02 ± 0.00 | 0.039 ± 0.000 | 16.2 ± 0.0 | 2.37 ± 0.02 |

For OPD(45-35-20) at 55 °C, the SAXS data fitted into a combined model which receives contribution from the lamellar model and a power law model. The scattering intensity *I(q)* can be described by:

|  | $I\left( q \right)=\frac{2\pi\varphi P_{l}\left( q \right)}{q^{2}\delta}+\frac{A}{q^{n}}+I_{bkg}$ | Eq. S6 |
| --- | --- | --- |

In the above equation, the first term describes the scattering from the lamellar structure (membrane of vesicles) and is the same as the model described by Eq. S3 and S4. But in this case, the polydispersity of *δ* was set to zero (uniform distribution) to improve the robustness of the combined model. The second term is the power law model, which is used to describe the scattering from the clusters. In this term, *A* is a multiplicative power-law scaling factor which is irrelevant to the volume fraction of the particles, and *n* is the power-law exponent.^5–7^

**Table S6.** Structure parameters of OPD(45-35-20) in 1 wt% H_2_O solution at 55 °C.

| **Sample Νο.** | ***T* (°C)** | ***φ*** | ***ρ* (×10^-6^ Å^-2^)** | ***δ* (nm)** | ***A* (×10^-6^)** | ***n*** |
| --- | --- | --- | --- | --- | --- | --- |
| OPD(45-35-20) | 55 | 0.003 ± 0.001 | 10.56 ± 0.14 | 36.2 ± 0.9 | 2.4 ± 1.0 | 2.8 ± 0.1 |

**Reference**

1. Constantinou, A. P. *et al.* Homo-and co-polymerisation of di(propylene glycol) methyl ether methacrylate-a new monomer. *Polym. Chem.* **12**, 3522–3532 (2021).

2. Feigin, L. A. & Svergun, D. I. *Structure Analysis by Small-Angle X-Ray and Neutron Scattering*. (Springer New York, NY, 1987).

3. Hammouda, B., Ho, D. L. & Kline, S. Insight into clustering in poly(ethylene oxide) solutions. *Macromolecules* **37**, 6932–6937 (2004).

4. Berghausen, J., Zipfel, J., Lindner, P. & Richtering, W. Influence of water-soluble polymers on the shear-induced structure formation in lyotropic lamellar phases. *J. Phys. Chem. B* **105**, 11081–11088 (2001).

5. Zhang, S. *et al.* Pore structure and gasification activity of coal and coke studied by small-angle X-ray scattering. *Philos. Mag. Lett.* **103**, (2023).

6. Bale, H. D. & Schmidt, P. W. Small-Angle X-Ray-Scattering Investigation of Submicroscopic Porosity with Fractal Properties. *Phys. Rev. Lett.* **53**, 596–599 (1984).

7. Ko, C. H. *et al.* Self-Assembled Micelles from Thermoresponsive Poly(methyl methacrylate)- b-poly(N-isopropylacrylamide) Diblock Copolymers in Aqueous Solution. *Macromolecules* **54**, 384–397 (2021).
